# Optimization of Processing Parameters and Quality Evaluation of Breakfast Flakes Prepared From Sonicated Corn and Its By‐Products Supplemented With Quinoa Powder

**DOI:** 10.1002/fsn3.71523

**Published:** 2026-02-12

**Authors:** Hina Niaz, Amna Javed, Rida Niaz

**Affiliations:** ^1^ Department of Nutrition and Dietetics The University of Faisalabad Faisalabad Pakistan; ^2^ College of Horticulture and Forestry Huazhong Agricultural University Wuhan China

**Keywords:** breakfast flakes, cob, corn grain, corn silk, quinoa, sonication, storage stability

## Abstract

Corn cob (C) and silk (S), produced in substantial quantities as agro‐waste, are rich in bioactive compounds with disease‐preventive potential. Thermal processing often leads to nutrient degradation. Therefore, this study explored the effect of optimized sonication—a non‐thermal technique with the potential to enhance the bioactive profile and stability—on Grain: G0, G10, G20, G30, Cob: C0, C10, C20, C30; and Silk S0, S10, S20, and S30, from which the best was incorporated into flakes. *C*_1_ (control) with 100% non‐treated corn grain flour (CGF), treatment (*T*_1_; 70:10, *T*_2_; 50:30, *T*_3_; 30:50, *T*_4_; 10 varied SCGF: SCF) and evaluated for moisture, bacterial, and fungal count in a storage period of 4 weeks. The significant result showed (G20 ≥ G0) for protein 15%, DPPH (340 ≥ 320) μg/g, TPC (1115 ≥ 1006) μg/g, and TFC increased 23 μg/g. In (C20), a significant difference was noted in fiber (34.6%–35.4%), TFC (1.05–1.85) μg/g, magnesium (1.88–1.98) μg/g, and vitamin A (188.7–193) μg/g. In S20, protein 2%, fiber 3%, DPPH 20 μg/g, TFC 190 μg/g, iron 6 μg/g, and vitamin A 1560 μg/g increased significantly. Storage of flakes exhibited lower moisture content (8%–10% reduction) and reduced microbial load, with bacterial and fungal counts remaining below detectable limits for 14 days, compared to control flakes which showed early microbial growth. Thus, the use of sonication and corn byproducts offers a cost‐effective, nutrient‐dense, diverse food product and ensures food security.

## Graphical abstract

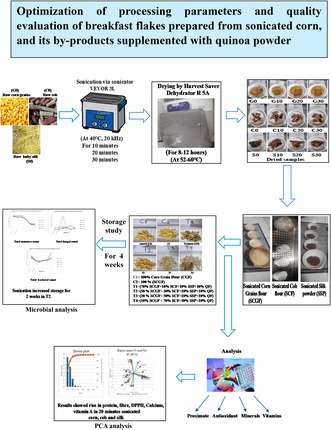

## 1 Introduction

Resource exploration is needed to accommodate the growing global population. By the end of 2050, there will likely be 9.7 billion people on the planet, placing a tremendous amount of strain on the agriculture industry (Khan et al. 2024). Simultaneously, considering the total amount of food harvested for human consumption (3900 million metric tons), it is estimated that over 1300 million metric tons of food are lost or abandoned annually worldwide (Uhlig et al. 2025). Along with that, in the modern era, a societal surge has been observed in different diseases like hypertension, hyperlipidemia, cardiovascular disease, and diabetes because of unhealthy food choices and a monotonous diet (Wang, Wang, Xiu, et al. 2024) (Godbharle 2024).

Corn ( *Zea mays* L.) ranked as the third most significant cereal crop, producing approximately 1.2 billion metric tons annually. Nutritionally, it promotes digestive tract health, reduces the risk of cardiovascular disease, type 2 diabetes, obesity, and several types of malignancy. Its dietary fiber plays a functional role in attenuating the postprandial glycemic response (Swapna et al. 2020). Corn is particularly a reliable source of iron, with an astounding 2.7 mg of iron per 100 g of grain, which helps to overcome anemia. It is also a source of the antioxidant thymine, which improves cognitive function. Additionally, it aids in the synthesis of acetylcholine, which improves memory (Baranowska 2023).

Owing to their convenience, palatability, and adaptability, corn‐based products, particularly cornflakes, have gained widespread acceptance as a preferred breakfast cereal across diverse populations worldwide as functional foods. It fulfills the demands by providing health benefits along with nutrition and reducing the risk of diseases. These are mostly served with liquid milk or sometimes consumed directly. These are made with many innovations in processing and raw materials, including corn, oats, rice, and the endosperm of wheat (Khuram and Rasul 2011).

The processing of corn generates substantial agricultural by‐products, including cob, husk, and silk, which are rich in nutrients and are often categorized as agro waste. These all are about 60%–70% of the total yield (Kaur, Singh, et al. 2023; Viroli et al. 2022). So, valorization of agro‐waste into functional food ingredients provides a dual solution by reducing food waste while enhancing dietary quality (Hossain et al. 2025). The challenges and opportunities associated with incorporating plant secondary metabolites into conventional pharmaceutical procedures, highlighting the importance of sustainable sourcing, formulation optimization, and regulatory considerations (Javed, Hashmi, Javaid, et al. 2025). To reduce these post‐harvest losses, overcome food insecurity, and increase shelf life, impoverished nations prioritize low‐cost technology. Although advanced food processors in wealthy nations place a higher priority on upcycling waste into valuable ingredients (Javed, Hashmi, Nadeem, et al. 2025). Also, different technologies are used to enhance the nutritional value, such as a suspended‐particle technology, spray drying finds extensive use in the food, pharmaceutical, and biotech industries (Amna Javed et al. 2018; Javed et al. 2019). Cob is utilized as a functional ingredient in gluten‐free products in the bakery and snacks such as muffins, breads, cakes, noodles, tortillas, and tacos.

There is a great deal of opportunity in the non‐thermal ultrasonication technique to convert corn waste biomass into a resource with additional value and newly developed products, just like spray drying technology, with extensive applications primarily in the food, pharmaceutical, and biotechnology industries (Lau 2018; Amna Javed et al. 2018). In ultrasonication, sound waves produced by ultrasound have frequencies higher than 20 kHz, which is outside the range of human hearing. Sonication conditions on the basis of literature are (40°C for 20 min), indicating that moderate temperatures and controlled exposure times are effective in improving functional and nutritional properties while minimizing nutrient degradation and thermal damage (Aday 2021). Water and energy waste are decreased because of the higher yield and faster extraction rate. Some researchers have studied sonication effects on the physiochemical properties of cereals and pseudocereals, including rice, black wheat, barley, sorghum, and millet. The result of some studies shows that ultrasonication enhances the extraction of polysaccharides, phenolic compounds, and other dietary ingredients; alters the structure of starch, influencing pasting qualities; and partially denatures proteins, enhancing their peptide availability and interfacial characteristics. All things considered, ultrasonication parameters offer a great deal of promise to enhance the transformation procedures and features of cereals and pseudocereals (Estivi et al. 2022). Nevertheless, corn and its by‐products are low in protein content. Since cereal proteins make up a sizable amount of dietary protein, they are essential to human nutrition. Cereal proteins show potential for a more environmentally friendly food industry as the demand for sustainable protein sources grows worldwide. So, the quinoa fortification of breakfast flakes is a good option to fulfill the requirement of protein and essential amino acids. Quinoa fortification is also safe for vegetarians because it is gluten‐free and can be used as a high‐quality substitute for dairy products (Kaur, Dhillon, and Mahajan 2023).

The processing techniques, thermal and non‐thermal, also significantly affect product shelf life during storage (Sattar et al. 2020). Moreover, previous studies have used edible coating to increase the shelf life (Farhan Saeed et al. 2017). However, depending on the food type and its sources, the processing parameters (frequency, amplitude, and treatment period) have changed, so in this study, ultrasonication is used to derive its effects on food quality. Because there is a need to step forward in using ultrasonication on food quality and shortage issues (Kenari and Razavi 2021). This study aimed to check the effect of optimized time of sonication on corn grains (G), cob (C), and silk (S) physicochemical, phytochemical, macro, and micronutrient qualities, the utilization of corn by‐products into flakes, and to study the storage of developed flakes.

## 2 Methodology

This study was divided into three phases. Phase I: In this phase, sonication of corn grains (CG), cob (C), and silk (S), along with chemical analysis of the samples, was done. Phase II: This involved the production of flakes. Phase III: This involved a storage study of developed flakes.

### 2.1 Procurement of Raw Material

Corn of local variety harvested at the physiological maturity stage, which is commonly used for cereal‐based product development, was collected from local fields in a village near Faisalabad using gloves and placed in plastic bags to be brought to the laboratory. The corn silk was separated from the cob, stalk, and peel. Grains were weeded out from the cob. Afterwards, this material was cleaned by hand to remove any foreign matter, dirt, and damaged material. Quinoa grains were obtained from the market. Then, all materials were stored in air‐tight zipper bags at room temperature, 25°C, for later use. The binding ingredients, salt, honey, and sugar, were purchased from the local market of Faisalabad. Chemicals used during research were purchased from Sigma Aldrich (Shahzad et al. 2022).

### 2.2 Phase I: Pretreatment With Sonication

The ultrasonication treatments were given by putting 100 g of corn, cob, and silk in 12 different small white color zipper bags of dimensions (20 cm × 10 cm) and closing them tightly so that water cannot enter the samples (Abadía et al. 2024). Three of them were non‐treated controls of corn grains (G0), cob (C0), and silk (S0). The remaining nine zipper bags were placed one by one in the water tub of the ultrasonic bath cleaner (VEVOR 3L) with a power of 20 kHz and times applied of 10, 20, and 30 min at 40°C. Ice was added to the water to maintain the temperature. Treatment of corn coded as G10, G20, G30; cob coded as C10, C20, and C30 and silk coded as S10, S20, and S30, respectively (Oladejo et al. 2022).

### 2.3 Standardized Drying Process Applied to Samples

**FIGURE 1:**
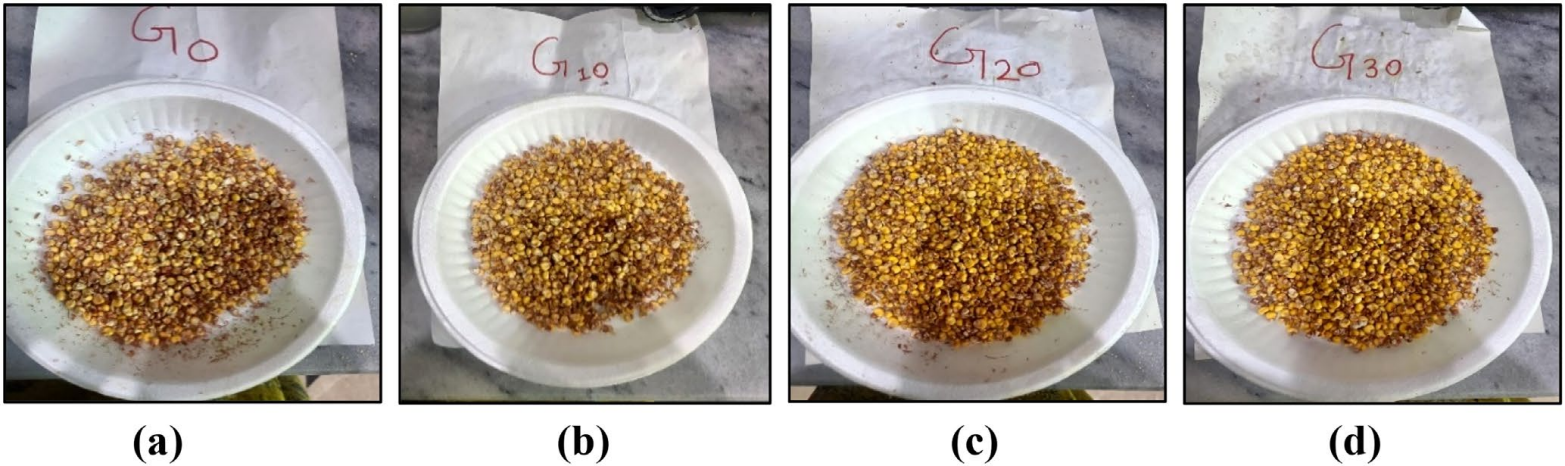
Pictorial presentation of sonicated corn grain samples after drying (a) G0, (b) G10, (c) G20, and (d) G30.

**FIGURE 2:**
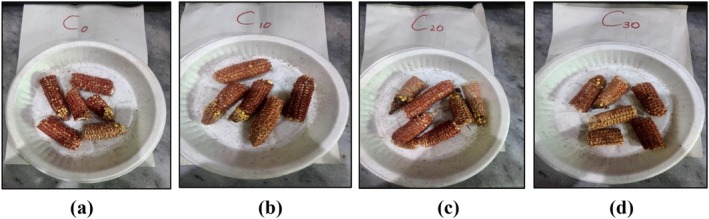
Pictorial presentation of sonicated cob samples after drying: (a) C0, (b) C10, (c) C20, and (d) C30.

**FIGURE 3:**
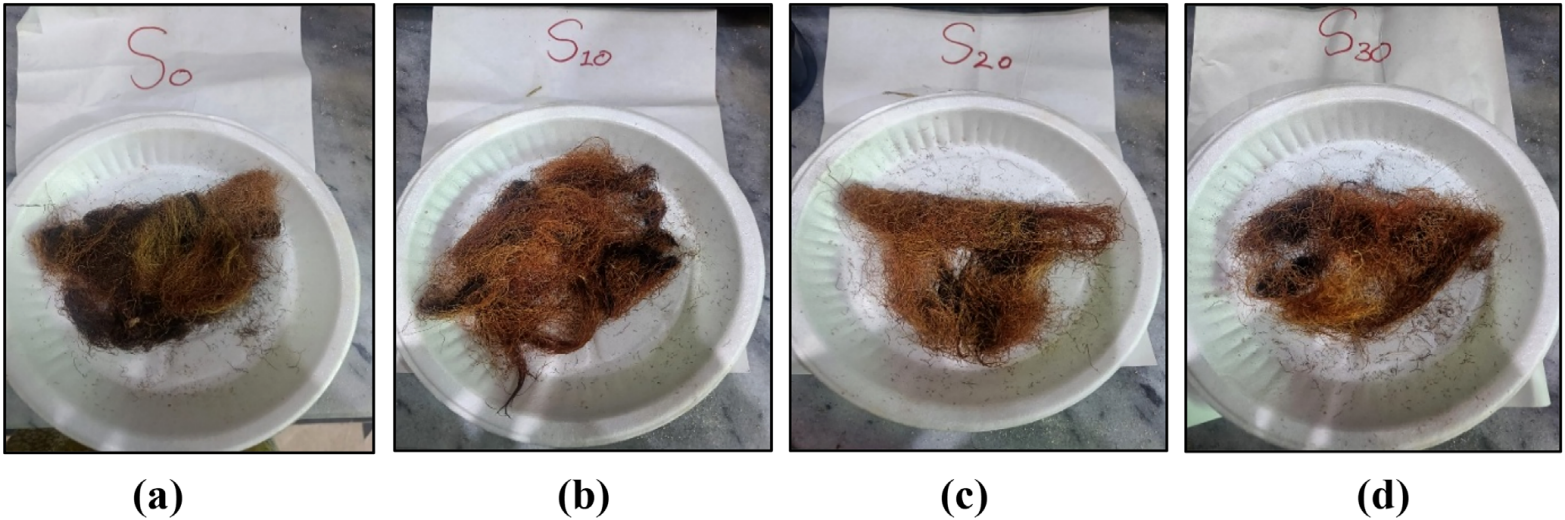
Pictorial presentation of sonicated silk samples after drying: (a) S0, (b) S10, (c) S20, and (d) S30.

Samples labeled as G0, G10, G20, G30, C0, C10, C20, C30, S0, S10, S20, and S30 illustrated in Table 1 were dried in a Harvest Saver dehydrator (Model R‐5A, USA) at 52°C–60°C for 8–12 h. Grains sample shown in Figure 1a–d, cob sample in Figure 2a–d, and silk in Figure 3a–d. Once dried, the samples were ground using a home grinder (WF‐9225 West Point) for 3–5 min and sieved through a 150‐mesh screen (particle size < 0.1 mm) to produce a fine powder. Then, they were again packed in 12 different small white color zipper bags with dimensions of (20 cm × 10 cm) and stored at room temperature, 25°C, for subsequent analysis (Muhammad Imran et al. 2016; Mujbaile et al. 2023; Salehi et al. 2024).

**TABLE 1:** List of abbreviations.

Abbreviations	Definition
CG	Corn grain
CGF	Corn grain flour
SCGF	Sonicated corn grain flour
C	Corn cob
SC	Sonicated cob
S	Silk
SSP	Sonicated silk powder
G0, G10, G20, G30	Corn grain samples were sonicated for 0, 10, 20, and 30 min, respectively
C0, C10, C20, C30	Corn cob samples were sonicated for 0, 10, 20, and 30 min, respectively
S0, S10, S20, S30	Corn silk samples were sonicated for 0, 10, 20, and 30 min, respectively
TPC	Total phenolic content
TFC	Total flavonoid content
RTE	Ready to eat
CFU	Colony‐forming units

### 2.4 Physicochemical Analysis

#### 2.4.1 Proximate Analysis

Proximate profile, including moisture, protein, fat, fiber, and ash analysis performed, on the same day according to AACC Dubat (2010).

##### 2.4.1.1 Moisture Percentage

The moisture content was determined by using a hot air oven (Method No. 44‐15.02) until the gain of constant weight (Buzgau et al. 2023) (D.O. 30‐1/02). The formula to calculate the moisture content was used:

$$\text{Moisture}\%=W_{1}–W_{2}/W_{1}\times 100$$

where *W*_1_ = weight of the sample before drying; *W*_2_ = weight of the dried sample.

##### 2.4.1.2 Ash Percentage

The total ash content was determined gravimetrically by incinerating pre‐weighed samples in a muffle furnace (NEY M‐525) at 550°C ± 2°C (AACC Method No. 08‐01.01). The ash content of the samples was calculated and expressed as a percentage on the basis of the fresh weight of the samples (International 2010).

$$\mathrm{Ash}\%=\text{Residual weight}\;\left(g\right)/\text{Weight of sample}\times 100$$

##### 2.4.1.3 Protein Determination

Protein content was estimated using the Kjeldahl method (AACC Method 46‐13.01) in the apparatus (KDY‐982), and nitrogen content was multiplied by 6.25 to calculate protein (International 2010). Nitrogen % = Vol. of 0.1 NH_2_SO_4_ used × (250 mL) × 0.0014 Weight of sample (g) × vol. of distillate taken

Protein%=Nitrogen%×6.25

##### 2.4.1.4 Fat Determination

Fat (%) was determined using Soxhlet extraction (R254S. Motherwell Way, Grays, United Kingdom) with n‐hexane as the solvent (AACC Method 30‐10.01; International 2010), and the formula was applied (International 2010)

$$\mathrm{Fat}\%=W_{2}-W_{1}/W\times 100$$

where *W* = Weight of the sample; *W*_1_ = Weight of the sample before extraction; *W*_2_ = Weight of the sample after extraction.

##### 2.4.1.5 Fiber Percentage

Fiber (%) was measured using acid–base digestion as described in AACC Method 32‐10.01, with final values corrected for ash content (International 2010; Paez et al. 2016)

$$\text{Fiber}\%=F_{1}–F_{2}/F_{0}\times 100$$

where *F*_0_ = Weight of the sample; *F*_1_ = Fiber plus ash content; *F*_2_ = Reweighed crucibles.

#### 2.4.2 Mineral Determination

Mineral concentration, including calcium, iron, magnesium, potassium, and zinc in the samples, was measured by AOAC Official (Method 995.13) using the protocol of atomic absorption spectroscopy after using standard solutions of varying strengths to standardize the instrument. Three separate runs of the samples were conducted on two different days (Method No 40‐70.01) (International 2010; Libron et al. 2021).

#### 2.4.3 Antioxidant Activity

Antioxidant activity, specifically free radical scavenging activity, was measured by using DPPH (2,2‐diphenyl‐1‐picrylhydrazyl) as the free radical source. A volume of 0.1 mL of the sample extract was mixed with 3.9 mL of 6 × 10^−5^ mol/L DPPH solution in methanol. After 30 min, the absorbance was measured at 515 nm, with methanol serving as the blank. The antioxidant activity was calculated using the following equation (Tul‐Muntaha et al. 2025).

$$\text{Antioxidant Activity}\%=\mathrm{Ab}\left(B\right)-\mathrm{Ab}\left(S\right)/\mathrm{Ab}\left(S\right)\times 100$$

where Ab(B) = Absorbance of the blank; Ab(S) = Absorbance of the sample.

#### 2.4.4 Total Phenolic Content (TPC)

The total phenolic compound in the sample was determined using the Folin–Ciocalteu reagent and gallic acid. A sample of 1–2 g is mixed with 1–2 mL of methanol before being centrifuged for 20 min at 5000 rpm. In a separate test tube, add 5 mL of 10% Folin–Ciocalteu reagent and 4 mL of 7% sodium carbonate. Mix well after 10 min. The test tubes were immersed in a water bath at 30°C–40°C ± 2°C for 40 min, then cooled. The sample optical density was measured at 760 nm with a spectrophotometer (Biowave II, 80‐3003‐75, Biochrom LTD, Cambridge, United Kingdom). The concentration was determined using the usual technique and the standard curve (Sharma 2024).

#### 2.4.5 Total Flavonoid Content (TFC)

The TFC of samples was measured by following the procedure defined briefly, as in a flask, 60 mL of 95% ethanol was combined with 3 g of sample, and the mixture was then extracted at 70°C in a water bath shaker. After one and a half hours, the extract was filtered using Whatman No. 1 filtered paper to get rid of the debris. To eliminate the solvent, the filtrate was evaporated. Before analysis, the residue was kept between 0°C and 4°C after being reconstituted with 5 mL of methanol. A spectrophotometer was used to perform spectrophotometric analysis (Sarepoua et al. 2015).

#### 2.4.6 Vitamin Analysis

Vitamin A (β‐carotene) was determined according to AOAC Official (Method 991.32), Vitamin B1 (Thiamine), vitamin K (phylloquinone), and vitamin E (tocopherol) content determination was carried out by spectrophotometer according to (Method No 86‐06.01) (International 2010; Topan et al. 2023).

### 2.5 Phase II: Development of Flakes

**FIGURE 4:**
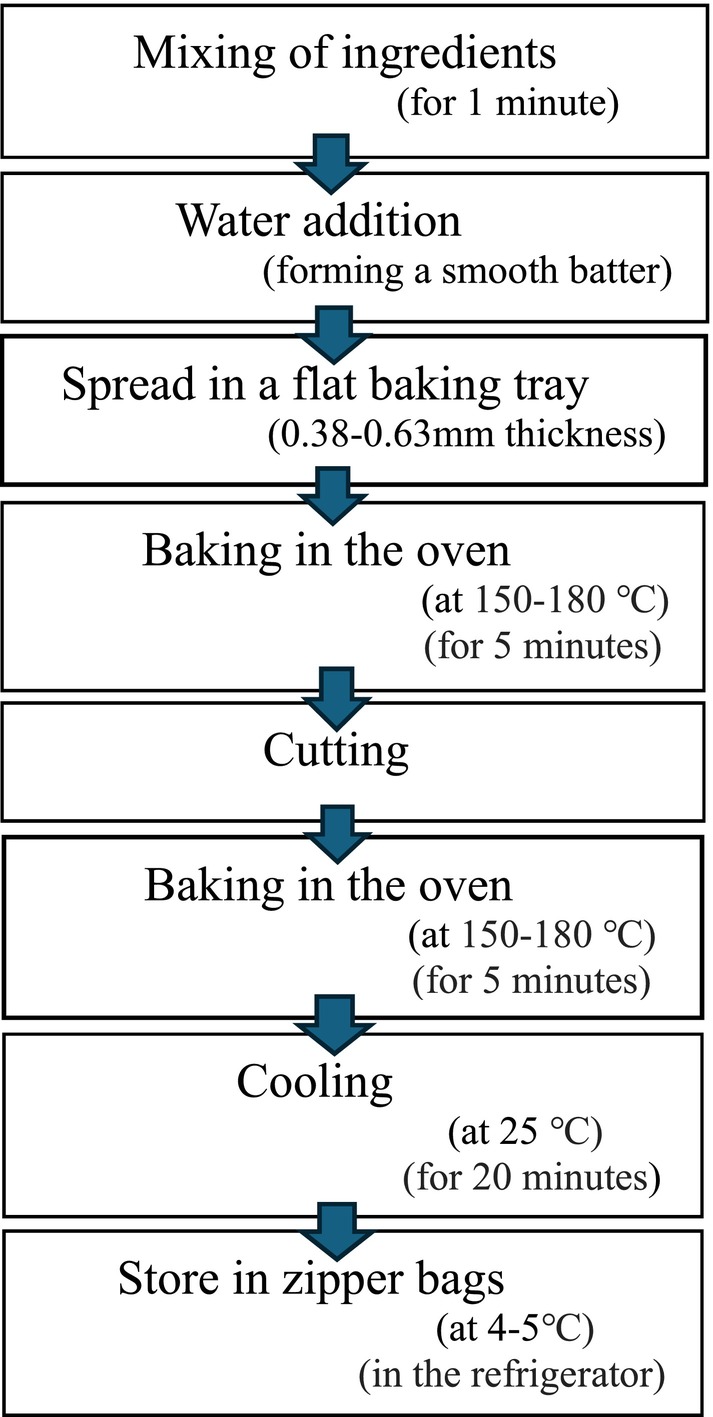
Development process of flakes prepared from sonicated‐based corn by‐products.

Flakes were developed by adding different proportions of 20‐min sonicated corn grain flour (SCGF), sonicated cob flour (SCF), sonicated silk powder (SSP), along with fortification of quinoa flour (QF) as given in Table 2. Two control groups were included: *C*_1_, which contained 100% untreated corn grain flour (CGF), and *C*_2_, which contained 100% SCGF. The other four treatments were followed as *T*_1_ (70% SCGF + 10% SCF); *T*_2_ (50% SCGF + 30% SCF); *T*_3_ (30% SCGF + 50% SCF); *T*_4_ (10% SCGF + 70% SCF), whereas SSP and QF were included in a constant amount of 10% in these four treatments, as shown in Table 3 (Dewidar and Hanan 2020). Formulations were mixed to form a homogeneous sample with other binding ingredients, including National salt (1 g), Adam white sugar powder (1–2 g), and then processed in an electric blender (WF 929 Westpoint) for 1 min. The amount of water was determined by preliminary trials according to the optimum batter consistency. The batter was placed in a food‐grade aluminum tray with a thickness of approximately 1–2 mm and baked at 150°C–180°C in a gas static oven (SG‐5000) for 5 min. At the end, the cutting was done and baked again at 150°C–180°C in the oven for 5 min to gain a crunchy texture, as illustrated in Figure 4 (El‐Waseif 2023).

**TABLE 2:** Code of material.

Ingredients	Code
Corn grain flour	CGF
Sonicated corn grain flour	SCGF
Sonicated cob flour	SCF
Sonicated silk powder	SSP
Quinoa flour	QF

**TABLE 3:** Composition of corn flake treatments.

Ingredient %	Treatments					
	Control 1	Control 2	*T*_1_	*T*_2_	*T*_3_	*T*_4_
CGF	100	—	—	—	—	—
SCGF	—	100	70	50	30	10
SCF	—	—	—	30	50	70
SSP	—	—	—	10	10	10
QF	—	—	—	10	10	10

### 2.6 Phase III: Storage Study

To determine the retention and stability of microbial, fungal, and moisture count in flakes. Each microbial assay was performed in triplicate (*n* = 3) for each sample to ensure accuracy and reproducibility of the results. All samples were stored in plastic zipper bags for 1, 2, 3, and 4 weeks at 4°C in a refrigerator. At the end of each week, the moisture content, total bacterial count (TBC), and total fungal count (TFC) were determined (Method No 35‐01.01) (Sánchez and Siche 2025).

#### 2.6.1 Total Moisture Content

The conventional procedures were used to determine the MC (moisture content) of samples. Two grams of each sample were weighed into moisture‐measuring vials and dried for one hour at 130°C. A sensitive analytical weighing scale was used to weigh the samples after they had been chilled in a desiccator (Okike et al. 2023).

#### 2.6.2 Total Bacterial Count

Total heterotrophic bacteria were counted by inoculating the surface of Nutrient Agar plates with 0.1 mL of 10^−3^–10^−5^ dilutions in triplicate. The aliquot was equally distributed with a sterile bent glass rod, and the infected plates were incubated in the incubator at 37°C for 24 h. Following incubation, colonies that formed on the respective plates were counted using the standard plate count to determine each sample's bacterial burden (Okike et al. 2023).

#### 2.6.3 Total Fungal Count

To measure the total heterotrophic fungal count, dextrose agar with antibiotics to prevent bacterial growth was utilized. The spread plate approach was used. An aliquot (0.1 mL) of 10^−3^ serially diluted samples was inoculated in duplicates over the surface of plates and evenly dispersed with a flame glass spreader. The plates were incubated at 25°C for 72 h, and the colonies were counted, with the mean count recorded (Okike et al. 2023).

### 2.7 Statistical Analysis

At least three determinations of an independent sample were used to display the results in mean ± standard deviation. The averaged data from the triplicate analyses were then subjected to an analysis of variance using one‐way and two‐way ANOVA, with Least Significant Difference (LSD) (*p* ≤ 0.05) applied to determine the significance of differences between treatments and storage time, as determined by IBM SPSS statistical software and Origin Pro 2021.

## 3 Results and Discussion

### 3.1 Proximate Profile of Sonicated Corn

**FIGURE 5:**
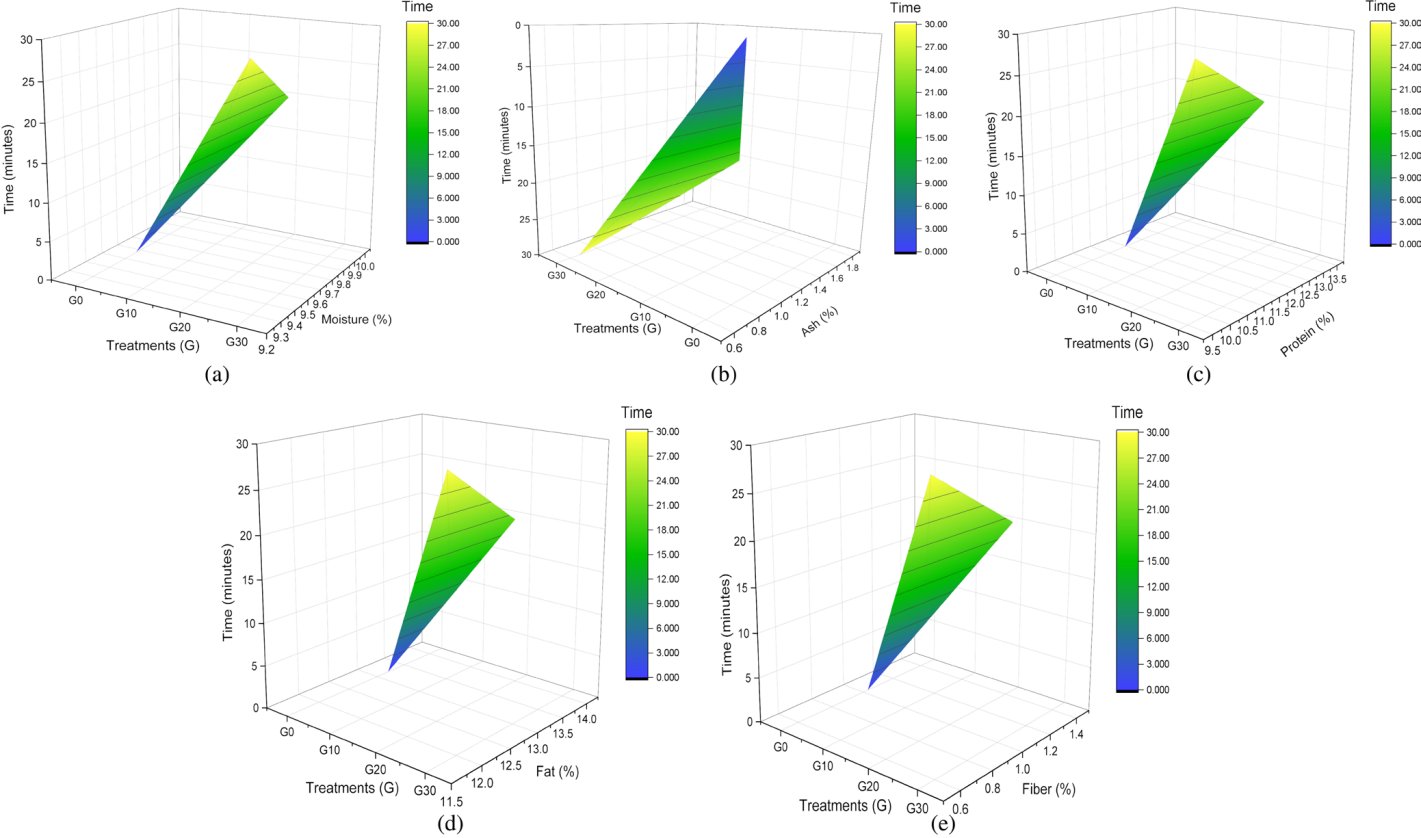
3D surface plot of corn grain (a) moisture, (b) ash, (c) protein, (d) fat, and (e) fiber.

Proximate composition of food is usually used for the quantitative analysis of food. The gradual increase in proximate composition of corn with time of sonication reached its maximum between 15 and 20 min, and afterward it started to decline, as presented in Table 4. Primarily, the moisture (%) level increased from 9.57% ± 0.05% to 9.97% ± 0.05%. These results support the principle that deeper hydration is made possible by the enhanced liquid penetration into the grains caused by ultrasonic waves as they travel through them, as outlined in finger millet malt's moisture content (%), which increased from 7.27% to 7.88% by the sonication process for 15 min (Adoni et al. 2025; Macho et al. 2020). Response surface plots of moisture in relation to sonication time duration are presented (Figure 5a). After soaking for a specific amount of time, the macro and micro components of the flour may drain and leach, resulting in a drop from the rootlets. However, ash (%) results slightly increase in G20, represented by plot (Figure 5b), which contradicts the literature, explained as the amount of ash in pulses was reduced by ultrasonication from 3.42 to 3.17 g/100 g (Banura and Singh 2023). Although protein (%) increased from sonication because of the breakdown of high‐molecular‐weight proteins into simple peptides and other constituents, as represented by (Figure 5c), up to 13.23% ± 0.23%, starting initially from 11.47% ± 0.52%. These findings are consistent with a previous study in which the protein content of control mung bean and lentil samples ranged from 19.96 to 23.86 g/100 g and 19.84 to 23.14 g/100 g, respectively. Following malted ultrasonication treatment, the protein content significantly increased, reaching values between 19.21 and 25.37 g/100 g for mung beans and 20.06 to 28.55 g/100 g for lentils (Banura and Singh 2023). Likewise, fat percentage changes from 13.20% ± 0.16% to 13.93% ± 0.05% in the G20 treatment shown by Figure 5d, proven by the study in which fatty acid (%) concentration in almond increases to a certain level when sonicated for 20 min as compared to 10 min sonication, including palmitic acid, oleic, and linoleic (Özcan et al. 2021) that is why ultrasonication‐assisted extraction offers the virtue of being a non‐destructive extraction method because it spares vital fatty acids, which could be utilized for their many health advantages. In the case of fiber ultrasonication of corn grains for 20 min, the highest 1.43% ± 0.09% fiber (%) is represented in (Figure 5e), after which it started to decline because sonication for longer periods also causes plant cell ruptures, releasing other chemicals that are likewise suspended in the extraction solvent and reducing its permeability. This has been reported in other studies, such as on optimizing the conditions aided by ultrasound to maximize the production of dietary fiber extracted from pumpkin sonicated for 23 min, which yields 16.0 ± 0.5 polysaccharides. The extraction yields steadily declined after this point (Martinez‐Solano et al. 2021).

**TABLE 4:** Proximate composition of sonicated corn.

Parameter (%)	G0	G10	G20	G30
Ash	0.8 ± 0.24^bc^	1.2 ± 0.08^b^	1.7 ± 0.21^a^	0.6 ± 0.24^c^
Moisture	9.5 ± 0.05^b^	9.7 ± 0.08^b^	9.9 ± 0.05^a^	9.2 ± 0.19^c^
Fat	13.2 ± 0.16^b^	13.5 ± 0.09^b^	13.9 ± 0.05^a^	11.8 ± 0.62^b^
Fiber	1.0 ± 0.05^a^	1.1 ± 0.09^b^	1.4 ± 0.09^a^	0.5 ± 0.33^b^
Protein	11.4 ± 0.52^b^	12.0 ± 0.41^b^	13.2 ± 0.21^a^	9.6 ± 0.47^c^

*Note:* Values presented as means ± standard error, *n* = 3. Values within each column followed by different letters (abcd) are significantly different at *p* ≤ 0.05. G = corn grains; G0 = 0 min; G10 = 10 min; G20 = 20 min; G30 = 30 min.

Moreover, a study on quinoa seeds reported that quinoa malt treated with 100% ultrasound intensity for 15 min and soaked for 6 h demonstrated the most favorable physicochemical properties (Eftekhari Yazdi et al. 2025). The findings of another study showed that when it came to preserving the proximate composition of three legume seeds, both conventional and innovative food processing methods acted differently. Although traditional food processing methods were found to have significantly higher (*p* ≤ 0.05) moisture and fat content, i.e., 28.30% and 1.40%, respectively, novel food processing methods sonication showed significantly higher (*p* ≤ 0.05) mean values for ash (4.16%), protein (21.34%), carbohydrate (50.10%), fiber (14.16%), and energy value (271.63 kcal/100 g) (Khan et al. 2025). These findings further support the potential of ultrasonication as a promising technique for industrial‐scale applications.

### 3.2 Phytochemical Contents of Sonicated Corn

**FIGURE 6:**
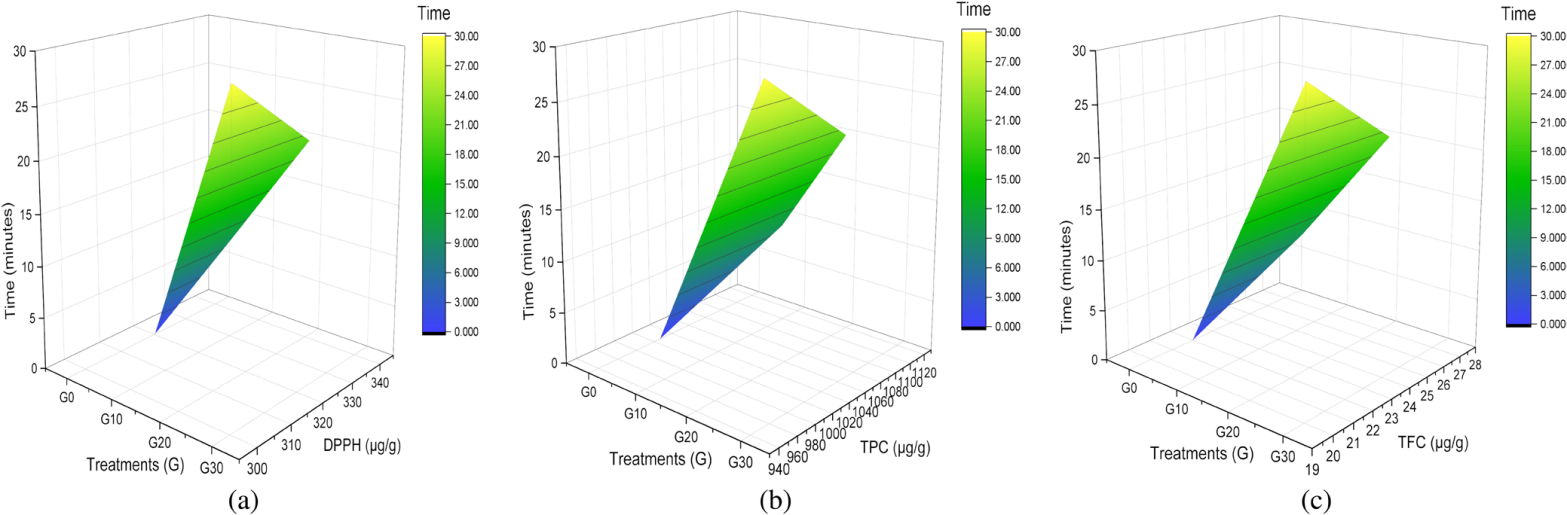
3D surface plot of corn grains antioxidant Profile (a) DPPH, (b) TPC, and (c) TFC.

The major contributors to the antioxidant activity are DPPH, phenolic, and flavonoid compounds. The changes in TPC, DPPH, and TFC of corn grains after sonication are summarized in Table 5. Sonication for 20 min resulted in a remarkable difference. The observed differences were statistically significant, as observed in the 3D plot (Figure 6a–c). In previous work, enhanced antioxidant activity (DPPH%) was reported from 33.36% ± 0.35% to 44.56% ± 0.22%, the extraction of TPC from 567.82 ± 0.91 to 595.58 ± 0.14, and TFC from 162.09 ± 0.23 to 235.64 ± 0.12 in finger millet and in barnyard millet flour. However, prolonged US results in degradation and cavitation, breaking down the cell wall and leading to the release of polyphenolic compounds (Bhandari et al. 2025). Primarily, according to the principle of cell wall breakdown and increased enzymatic activity during germination, enhanced antioxidants by 35%, and dietary fiber by 20%, by application of ultrasonication during the soaking of amaranth was reported (Awasthi et al. 2025). An increase in free phenolic acid content by liberating bound phenolics from sorghum flour by ultrasonication was observed when the impact of various process parameters on specific energy was investigated during both batch and continuous flow ultrasonication (Lohani and Muthukumarappan 2021). Along with that, researchers investigated the impact of a continuous‐loop ultrasonic system on the enzymatic activity, phenolic profile, antioxidant activity, color, and bio accessibility of açai pulp for better processing and health benefits. Continuous processing increased the flavonoid content up to 452 mg of GAE per 100 g. The continuous‐loop ultrasonic technology produced high‐quality açai pulp with shorter processing periods and better nutritional and sensory qualities (Dantas et al. 2025). Another study showed that utilizing 30 min for ultrasonication and 72 h for germination at 40°C produced the best results for creating ultrasonicated and germinated kodo and tiny millet flour with the maximum antioxidant activity and the lowest antinutrient content (phytate and tannin) (Dey et al. 2023). These findings underscore the significance of employing non‐thermal processing techniques in enhancing the extraction and preservation of antioxidant compounds from these by‐product plant matrices.

**TABLE 5:** Antioxidant of sonicated corn samples.

Treatments (μg/g)	G0	G10	G20	G30
DPPH	320.2 ± 0.82^b^	330.6 ± 0.16^b^	340.3 ± 0.22^a^	300 ± 0.65^c^
TPC	1006 ± 1.25^b^	1099 ± 0.82^b^	1115 ± 8.34^a^	952.6 ± 33.48^c^
TFC	22.0 ± 1.63^b^	25.3 ± 0.47^b^	27.3 ± 0.94^a^	20.0 ± 0.16^b^

*Note:* Values presented as means ± standard error, *n* = 3. Values within each column followed by different letters are significantly different at *p* ≤ 0.05. G = corn grains; G0 = 0 min; G10 = 10 min; G20 = 20 min; G30 = 30 min.

### 3.3 Mineral Content of Sonicated Corn

**FIGURE 7:**
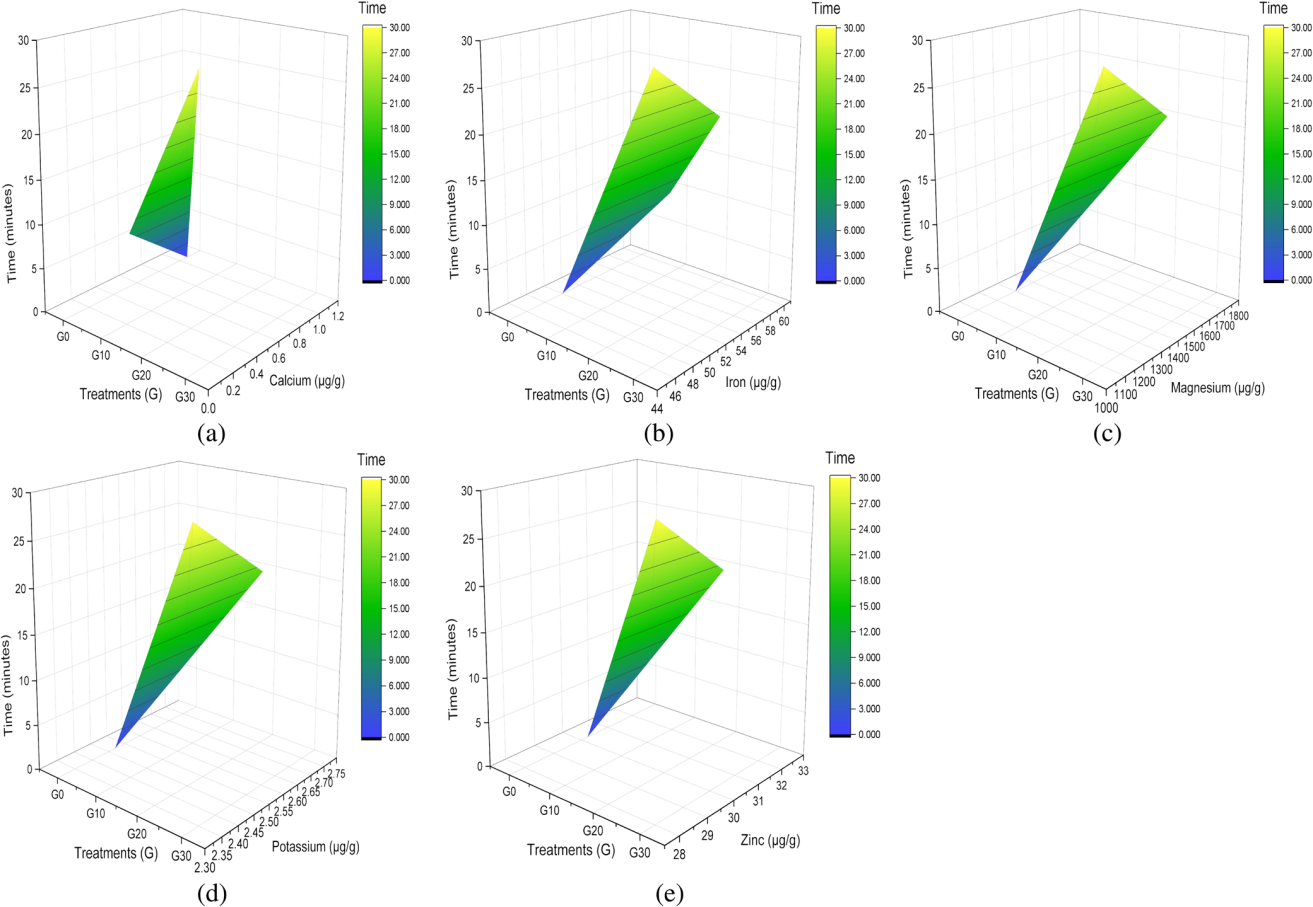
3D surface plot of minerals in corn grain: (a) calcium, (b) iron, (c) magnesium, (d) potassium, and (e) zinc.

The mineral concentration of the corn grain detected is presented in Figure 7a–e, along with the time, which fluctuates. The calcium level has fallen from 1.07 μg/g to 0.10 μg/g at 20 min. Although the proportions of iron, magnesium, potassium, and zinc increase by 1%–2% at 20 min. A significant level of values in the form of (*p* ≤ 0.05) as exhibited in Table 6, respectively. This is consistent with the results of a research study that reported sonication of finger millet for 18 min enhanced the iron (mg/100 g) from 3.73 ± 0.54 to 7.12 ± 0.22, except for the calcium level, which contradicts (Adoni et al. 2025). Sonication improved the permeability of seed cell walls and encouraged interaction between iron cations and polyphenols, resulting in a complex that increases iron stability and bioavailability. Additionally, it is explained by the fact that the sonication process increases the concentration of free calcium after dissolving divalent metal ions (such as calcium, iron, and zinc) that bind to proteins. Magnesium, potassium, and zinc proportions (μg/g) at their peak were 1730 ± 0.19, 2.70 ± 0.13, and 32.33 ± 0.24 at 20 min. These results are parallel to the study except for the calcium level reported when the ultraviolet (UV) for 30 min and sonication (US) for 10 min‐combination was used as a pretreatment, high mineral contents Ca, Fe, K, Mg, and Zn (119.6%, 21.4%, 77.6%, 15.4%, and 42.6%) were achieved in black lentils (Levent and Aktaş 2024).

**TABLE 6:** Mineral content of the sonicated corn sample.

Minerals (μg/g)	G0	G10	G20	G30
Calcium	1.07 ± 0.09^a^	0.23 ± 0.09^bc^	0.37 ± 0.02^b^	0.10 ± 0.15^c^
Iron	50.0 ± 1.63^b^	58.6 ± 0.47^d^	59.6 ± 0.47^a^	46.0 ± 0.05^c^
Magnesium	1300 ± 0.08^bc^	1480 ± 0.03^ab^	1730 ± 0.19^a^	1100 ± 0.08^c^
Potassium	2.47 ± 0.05^c^	2.57 ± 0.05^b^	2.70 ± 0.13^a^	2.33 ± 0.05^d^
Zinc	30.2 ± 0.29^c^	31.0 ± 0.09^b^	32.3 ± 0.24^a^	28.0 ± 0.01^d^

*Note:* Values presented as means ± standard error, *n* = 3. Values within each column followed by different letters are significantly different at *p* ≤ 0.05. G = corn grains; G0 = 0 min; G10 = 10 min; G20 = 20 min; G30 = 30 min.

### 3.4 Sonication Effect on Corn Vitamins

**FIGURE 8:**
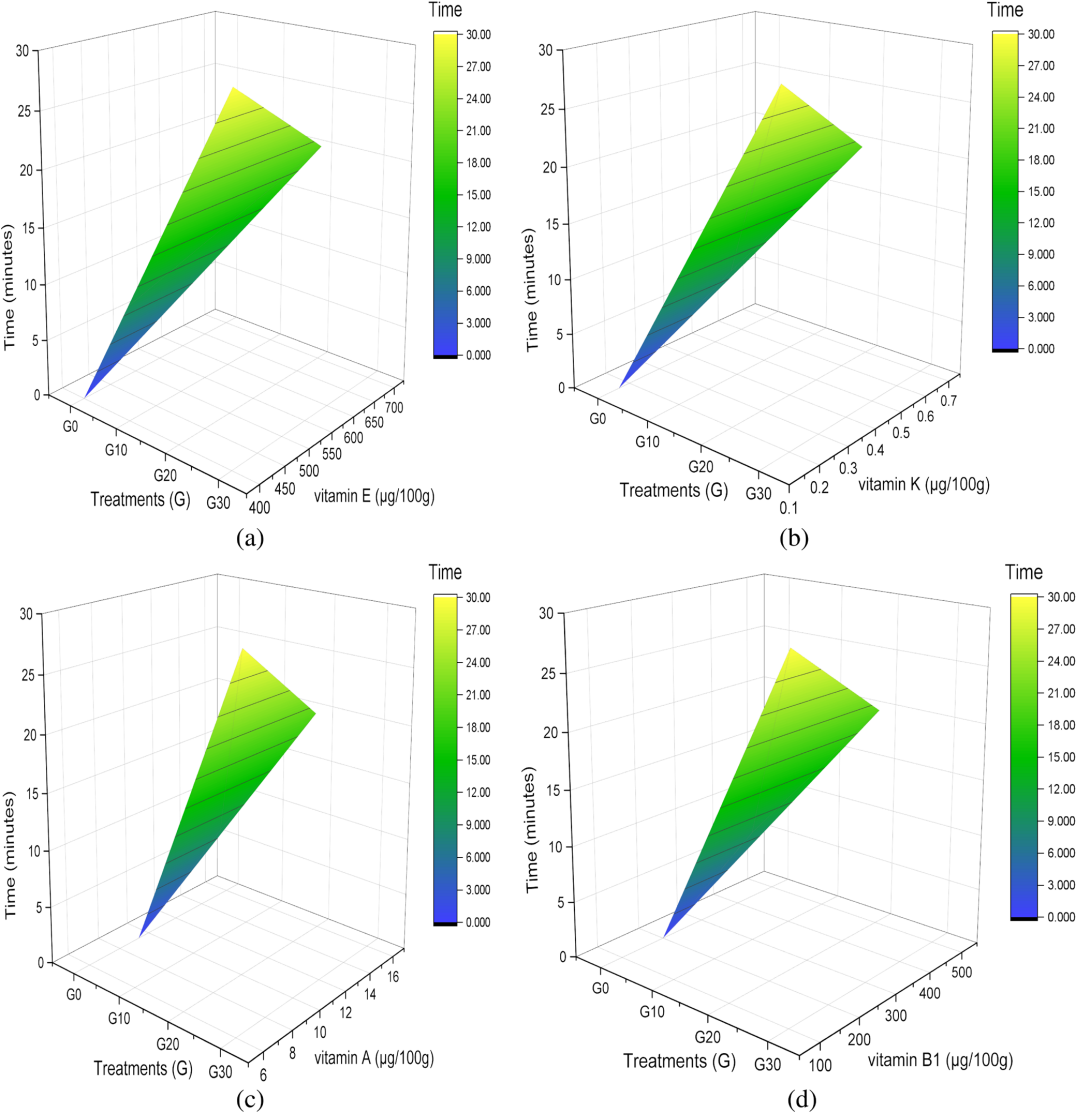
3D surface plot of corn grains vitamins (a) vitamin E, (b) vitamin K, (c) vitamin A, and (d) vitamin B1.

In the corn grain sample, the highest response of micronutrients is illustrated in Figure 8a,d, subjected to sonication for 20 min. A comparison of significant values with the control treatment (G10) and 30 min (G30) is presented in Table 7. The notable change in retention of vitamin E, vitamin K, vitamin A, and vitamin B1 concentrations is observed at 20 min. This is paired with the other study of researchers about the application of sonication on maize seedlings compared to the untreated maize seedlings; the carotenoid content rose and peaked at 8.21 ± 0.44 μg/g fresh weight (FW) after 8 min of ultrasonic therapy. However, tocopherol first decreased but then started to climb (258.1 ± 6.4 μg/g FW). Because sonication pretreatment altered the dominant tocochromanol component, shifting from γ‐tocotrienol to α‐tocopherol, with α‐tocopherol content being 1.3 times higher than in the control group (Zhang et al. 2023). In the case of vitamin K in Figure 8b, its stability to moisture and air results in better efficiency. The longer the ultrasonic duration, the concentration gradually increased, which is also observed in our results at 20 min. The results are justifiable, and parallel to another study in which vitamin K1 peak area peaked at 50 min and subsequently leveled out when extracted from different foods (Xu et al. 2020). Moreover, a rise in the concentration of vitamin B1 observed in Figure 8c aligned with research of other scientists on sonicated samples of dietary supplements that had higher mean levels of cyanocobalamin than their heat‐extracted counterparts. A 15‐min extraction in an ultrasonic bath was sufficient to liberate cyanocobalamin before its quantitative measurement (Chandra‐Hioe et al. 2020). Corn and fermented foods have also shown an increase in carotenoids as a result of the ultrasonication and fermentation procedures (Suo et al. 2022).

**TABLE 7:** Vitamin profile of the sonicated corn sample.

Vitamins (μg/100 g)	G0	G10	G20	G30
Vitamin E	400 ± 0.004^c^	400 ± 0.01^b c^	700 ± 0.01^b^	400 ± 0.04^c^
Vitamin K	0.166 ± 0.04^b^	0.38 ± 0.02^a^	0.67 ± 0.08^a^	0.16 ± 0.04^c^
Vitamin A	9.3 ± 0.4^bc^	12 ± 0.8^b^	15.6 ± 0.47^a^	6.33 ± 0.47^c^
Vitamin B1	200 ± 0.01^c^	300 ± 0.01^b^	500 ± 0.01^a^	100 ± 0.04^d^

*Note:* Values presented as means ± standard error, *n* = 3. Values within each column followed by different letters are significantly different at *p* ≤ 0.05. G = corn grains; G0 = 0 min; G10 = 10 min; G20 = 20 min; G30 = 30 min.

### 3.5 Impact of Sonication on Cob Nutrients

**FIGURE 9:**
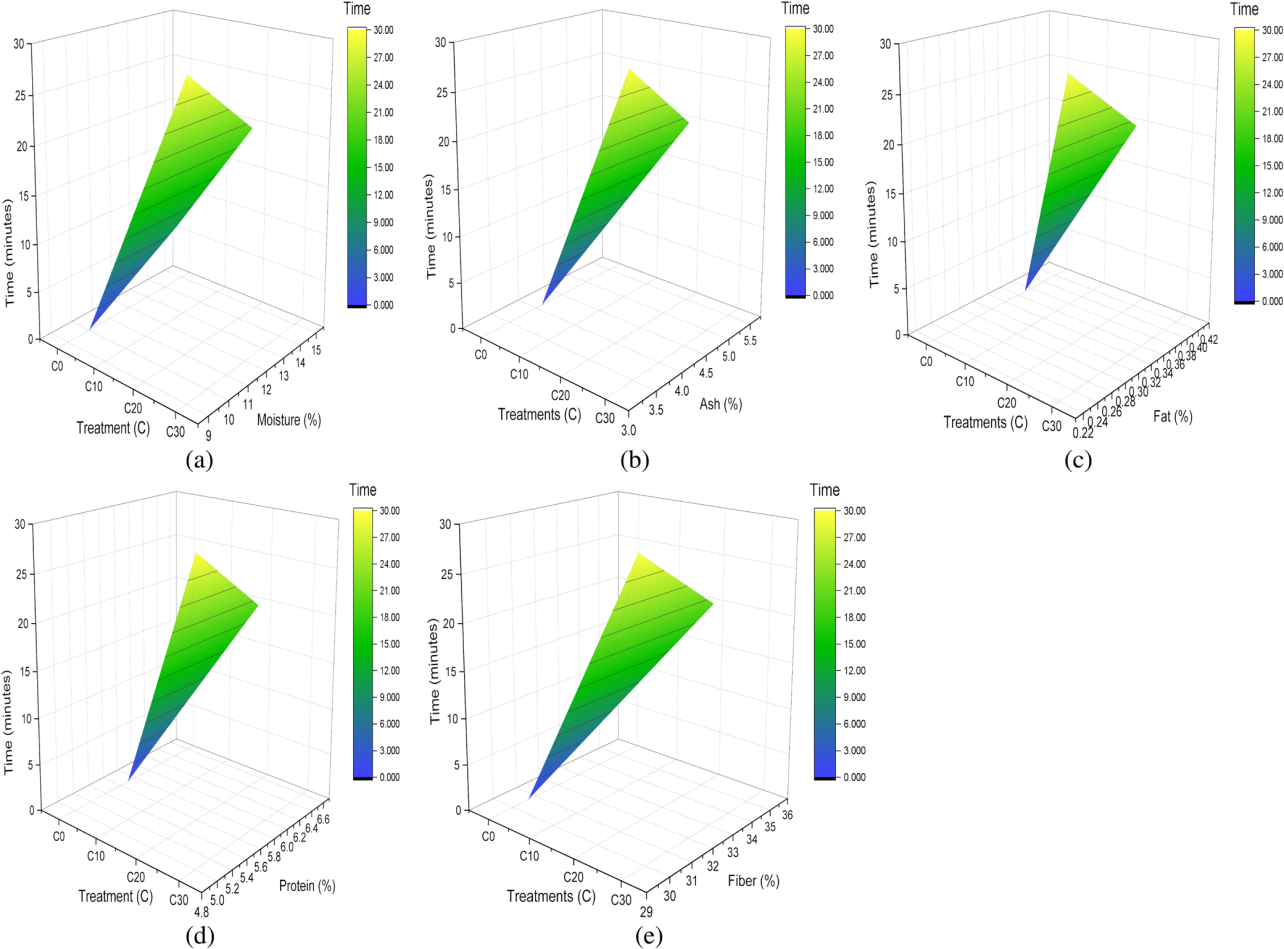
3D surface plot of cob: (a) moisture, (b) ash, (c) fat, (d) protein, and (e) fiber.

Significance values for ash, moisture, protein, fat, and fiber after analysis of samples are depicted in Table 8. Moisture content increases after sonication by about 2%–4% shown in Figure 9a, because of liquid water seeping into the sample during the soaking period. This is justified as the moisture content was studied on sonicated carrot air drying, in which the initial moisture content, moisture diffusivity changed from 8.60 kg water/kg dry solid to 8.97 kg water/kg dry solid, from 43% to 90% at 20 min. However, differences in the evolution of moisture content over 30 to 60 min were insignificant (Tran et al. 2023; Vela et al. 2021). Ash content changes about 1% as elaborated by Figure 9b, which is supported by the African yam bean (AYB) flour study (Olaitan et al. 2025), which was affected by bioprocessing and ultrasonication (US) treatment for 15 and 30 min (US 15 and US 30). This indicates ash contents rose from 2.02 ± 0.05 to 2.71 ± 0.05. However, in the same study, the decline in fat contents (%) does not support our results, which range from 0.35% ± 0.01% to 0.40% ± 0.01%, as given in Figure 9c. The effect of sonication time on protein content of cob reported a negligible rise about 0.5% from 10 to 20 min after which it starts to fall as given in Figure 9d that is opposite to literature as reported, in yellow cassava it starts to decline from 10 min with distilled water (UDW) and osmotic dehydration (UOD) at 20 min reason described as combined effects of ultrasound and osmotic concentration caused the protein content to degrade. There was an increase in fiber (%) content observed in the same study (Oladejo et al. 2022) that aligned with our results, represented by Figure 9e. The cause of these discrepancies may be the variety, sonication time, solvent type, and some analytical measurements.

**TABLE 8:** Chemical composition of sonicated cob.

Parameter (%)	C0	C10	C20	C30
Ash	4.10 ± 0.05^c^	4.33 ± 0.12^b^	5.50 ± 0.41^a^	3.4 ± 0.33^d^
Moisture	10.0 ± 0.82^c^	12.6 ± 0.9^b^	14.6 ± 0.4^a^	9.0 ± 0.82^d^
Fat	0.35 ± 0.01^a^	0.37 ± 0.01^a^	0.40 ± 0.01^a^	0.2 ± 0.05^b^
Fiber	30.6 ± 0.4^c^	32.0 ± 0.8^b^	35.5 ± 0.4^a^	29.6 ± 0.4^c^
Protein	5.70 ± 0.04^b^	6.0 ± 0.16^ab^	6.5 ± 0.41^a^	5.0 ± 0.08^c^

*Note:* Values presented as means ± standard error, *n* = 3. Values within each column followed by different letters are significantly different at *p* ≤ 0.05. C = Corn cob; C0 = 0 min; C10 = 10 min; C20 = 20 min; C30 = 30 min.

### 3.6 Changes in Phytochemical Content of Cob Induced by Sonication

**FIGURE 10:**
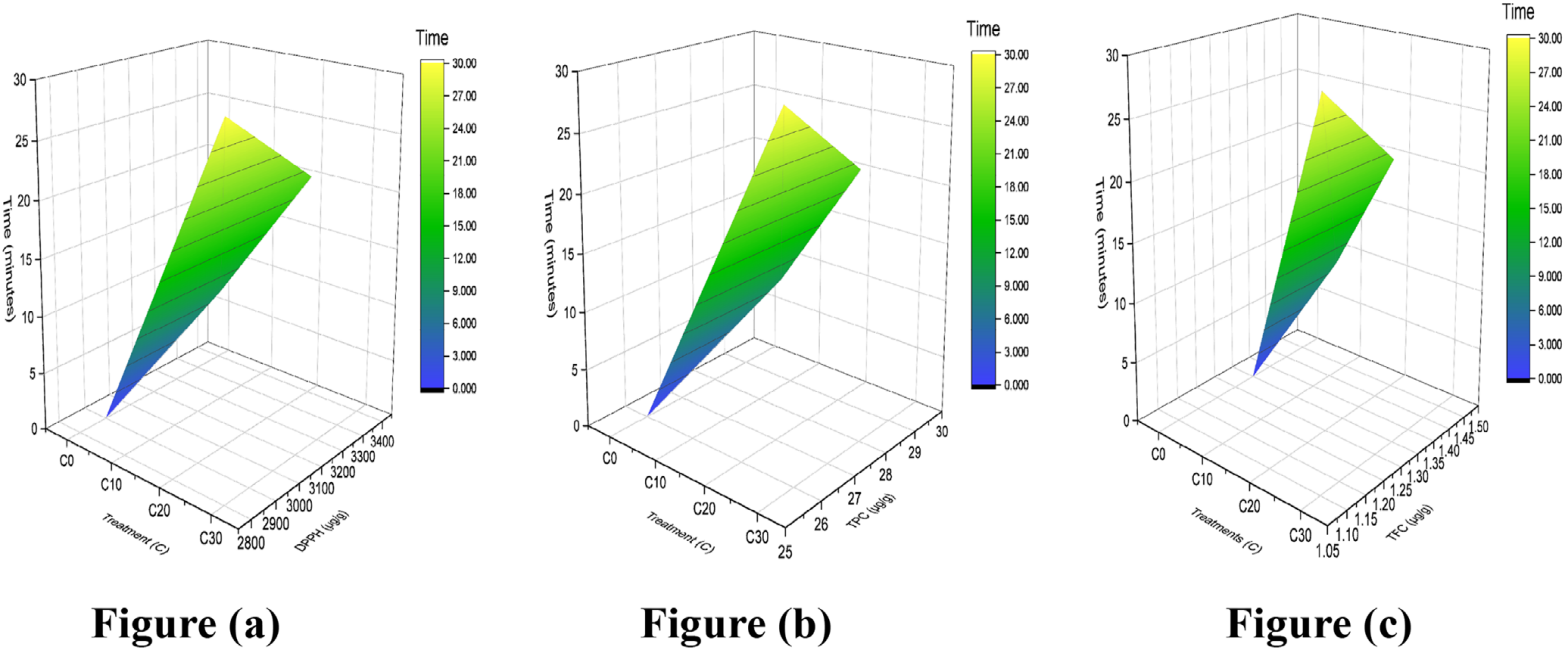
3D surface plot of the cob antioxidant profile: (a) DPPH, (b) TPC, and (c) TFC.

In the cob, the change in dynamics of phytochemical concentration with time of sonication is depicted in (Figure 10a–c) of a surface plot. The notable improvement in C20, elaborated by significant values DPPH, TPC, and TFC 3400 ± 0.4^a^, 1.45 ± 0.4^a^, and 29.67 ± 0.4^a^ given in Table 9, is supported by other scientists' study on the effect of extraction time on the total anthocyanin content (TAC) from corn cob. As the extraction time increased from 5 to 10 min, the TAC exhibited a slight, non‐significant increase. However, a further increase in extraction time to 15 min resulted in a significant decrease in TAC (Piyapanrungrueang et al. 2016). Additionally, the ultrasound‐assisted extraction of anthocyanins and TPC from dried purple waxy corn cobs under optimal conditions, specifically an extraction temperature of 65°C for 30 min, yielded the maximum amounts of anthocyanins and total phenolic compounds (Muangrat et al. 2018). Similarly, the optimal conditions identified in a study on efficient extraction of TPC, ellagic acid, gallic acid, punicalin, and punicalagin from pomegranate peel were 25 min, an ethanol concentration of 59%, a solid‐to‐solvent ratio of 1:44, and an extraction temperature of 80°C (Živković et al. 2018). This reinforces that such processing methods effectively maximize the antioxidant potential while minimizing nutrient degradation, highlighting their value in sustainable food processing and functional ingredient development.

**TABLE 9:** Antioxidant profile of the sonicated corn cob sample.

Parameter (μg/g)	Control	C10	C20	C30
DPPH	2900 ± 0.4^b^	3200 ± 0.4^b^	3400 ± 0.4^a^	2800 ± 0.8^c^
TFC	1.30 ± 0.08^b^	1.42 ± 0.4^b^	1.45 ± 0.4^a^	1.10 ± 0.08^c^
TPC	26.0 ± 0.8^b^	28.6 ± 0.4^b^	29.6 ± 0.4^a^	25.6 ± 0.4^b^

*Note:* Values presented as means ± standard error, *n* = 3. Values within each column followed by different letters are significantly different at *p* ≤ 0.05. C = Corn cob; C0 = 0 min; C10 = 10 min; C20 = 20 min; C30 = 30 min.

### 3.7 Sonication Impact on Cob Mineral Concentration

**FIGURE 11:**
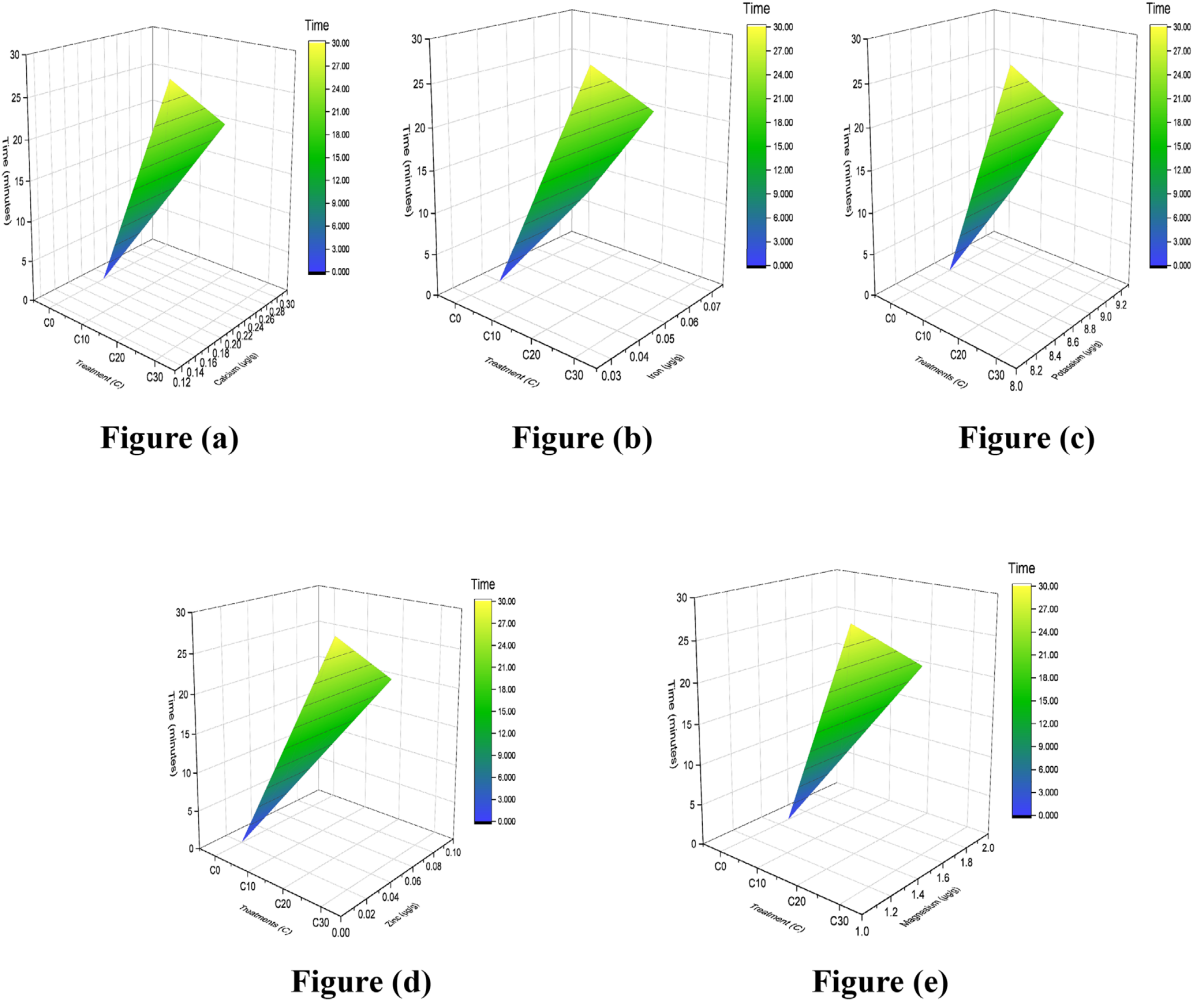
3D surface plot of minerals in cob: (a) calcium, (b) iron, (c) potassium, (d) zinc, and (e) magnesium.

Individually for all parameters, the 3D surface plots in Figure 11 reveal that with increasing time, there is a shift in mineral concentration (μg/g): calcium 0.09, iron 0.03, magnesium 0.46, potassium 0.53, and zinc 0.07. However, significant treatment with the help of a small letter is shown in Table 10. Results commensurate with the study of (Akbar et al. 2025), in which the mineral profile of baby corn cob powder reveals significant differences in the concentrations of calcium, magnesium, zinc, phosphorus, and iron on the basis of particle size after using a probe sonicator for 20 min. A clear trend was observed, where the levels of these minerals increase as the particle size reduces from over 500 to 53 μm—specifically, calcium, magnesium, zinc, phosphorus, and iron content increase. Furthermore, the nutritional index of free amino acids and the in vitro bio accessibility of calcium and iron were significantly enhanced after investigating the impact of an innovative processing method that integrates HIU stimulation (28 kHz, 17.83 W cm^−2^) during the pre‐germination phase (Gunathunga et al. 2024; Xia et al. 2020). When fermentation and ultrasonic treatment were combined, soybeans' antioxidant activity increased, as reported in a study (Kong et al. 2023).

**TABLE 10:** Mineral content of the sonicated cob sample.

Parameter (μg/g)	Control	C10	C20	C30
Calcium	0.19 ± 0.03^b^	0.23 ± 0.02^b^	0.28 ± 0.02^a^	0.13 ± 0.01^c^
Iron	0.04 ± 0.01^c^	0.06 ± 0.01^b^	0.07 ± 0.01^a^	0.03 ± 0.01^c^
Magnesium	1.47 ± 0.08^b^	1.60 ± 0.01^b^	1.93 ± 0.05^a^	1.07 ± 0.09^c^
Potassium	8.60 ± 0.17^b^	8.94 ± 0.04^a^	9.13 ± 0.09^a^	8.13 ± 0.09^c^
Zinc	0.02 ± 0.01^b^	0.03 ± 0.07^b^	0.09 ± 0.01^a^	0.01 ± 0.01^b^

*Note:* Values presented as means ± standard error, *n* = 3. Values within each column followed by different letters are significantly different at *p* ≤ 0.05. C = Corn cob; C0 = 0 min; C10 = 10 min; C20 = 20 min; C30 = 30 min.

### 3.8 Sonication Effect on the Cob Vitamin Profile

**FIGURE 12:**
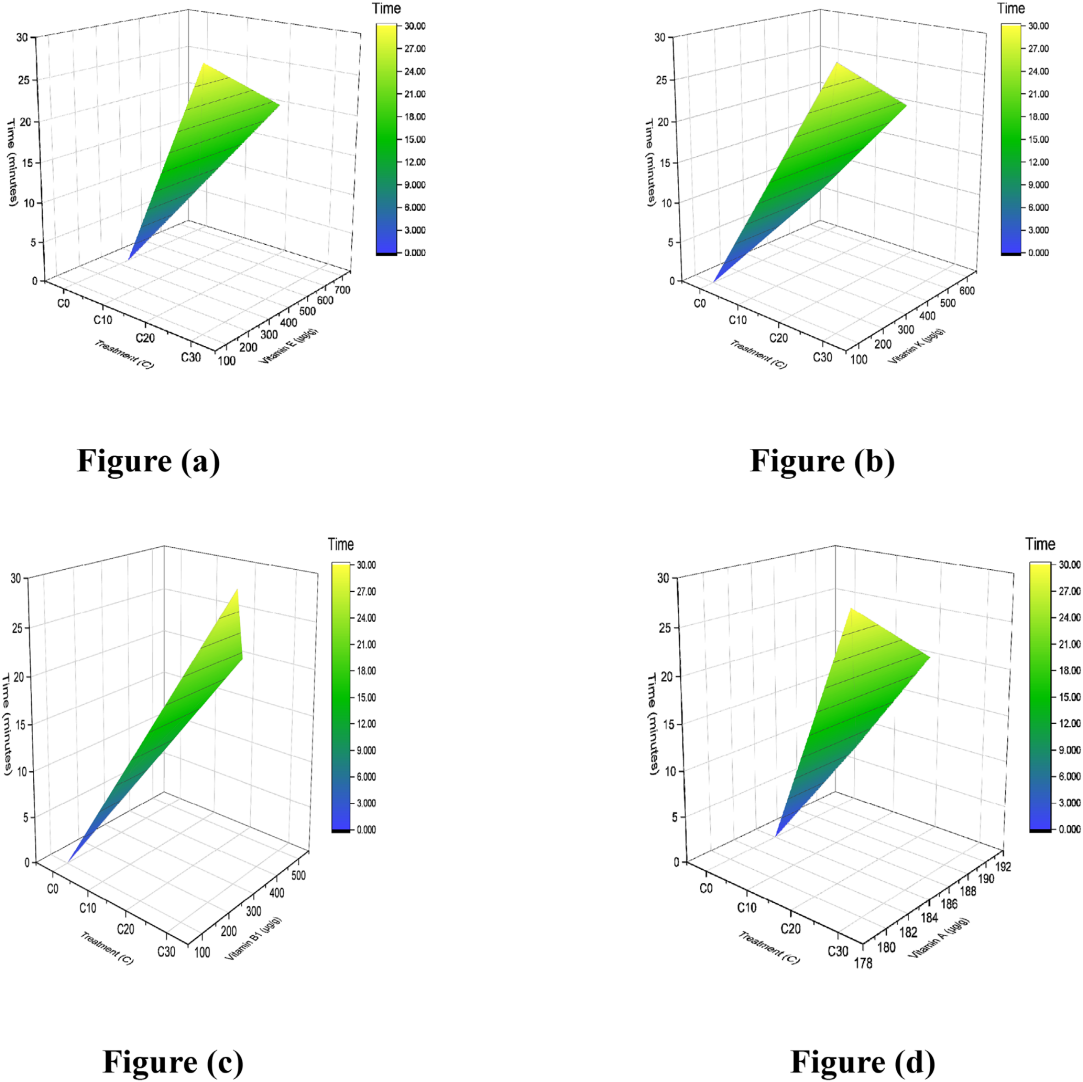
3D surface plot of cob vitamins: (a) vitamin E, (b) vitamin K, (c) vitamin B1, and (d) vitamin A.

The kinetics for vitamin E, K, A, and B1 proportions showed an increase (μg/g) up to 700 ± 0.2, 600 ± 0.04, 191 ± 0.02, and 500 ± 0.0 in C20 by the plots in Figure 12. In comparison to the control treatment, Table 11 shows a significant treatment effect, backed by the study reporting the extraction of carotenoids from sea buckthorn pomace (SBT‐P) using ultrasonication for 30 min and microwave‐assisted extraction (MAE). These techniques were combined with natural green solvents, specifically corn oil and olive oil. The total carotenoid content (TCC) obtained from SBT‐P using UAE and MAE ranged from 26.91 ± 1.96 to 34.35 ± 0.94 mg per 100 g (Sharma et al. 2022). The recovery yield of flavonoid, terpenoid (precursor of vitamin E), vitamin C, and reducing sugar contents from yacon roots was investigated under ideal circumstances. At 20 min, yields were 230.39 mg D‐glucose equivalents per gram, 107.78 mg ascorbic acid per gram, 47.93 mg ursolic acid equivalents per gram, and 7.95 mg rutin equivalents per gram. Ultrasonic‐microwave‐assisted extraction is thought to be a sustainable and efficient way to recover target compounds from yacon roots, since it demonstrated noticeably better performance than single‐extraction techniques (Vo et al. 2024). Because of vitamin K1's stability to moisture and air, the longer the ultrasonic duration, the better the extraction efficiency. It is reported in research that the vitamin K1 peak area peaked at 50 min of sonication and gradually leveled out in rapeseed, soybean, peanut, and sesame oilseeds (Xu et al. 2020). Sonication therapy enhanced the availability of provitamin A, vitamins B3, B5, C, and E by freeing them from the apoenzymes to which they are attached. Results of other studies proved that the changes in juice quality metrics caused by variations in ultrasonic power density, processing duration, and temperature, as well as the improvement of juice homogeneity via 15 min of sonication (Santos et al. 2018).

**TABLE 11:** Vitamin profile of the sonicated cob sample.

Parameter (μg/g)	Control	C10	C20	C30
Vitamin E	340 ± 0.04^a^	500 ± 0.08^b^	700 ± 0.2^a^	100 ± 0.04^a^
Vitamin K	100 ± 0.04^a^	400 ± 0.04^a^	600 ± 0.04^a^	100 ± 0.04^a^
Vitamin A	184 ± 0.4^a^	188 ± 0.8^ab^	191 ± 2.3^a^	179 ± 0.4^a^
Vitamin B1	100 ± 0.04^a^	200 ± 0.09^b^	500 ± 0.04^a^	300 ± 0.04^a^

*Note:* Values presented as means ± standard error, *n* = 3. Values within each column followed by different letters are significantly different at *p* ≤ 0.05. C = Corn cob; C0 = 0 min; C10 = 10 min; C20 = 20 min; C30 = 30 min.

### 3.9 Sonicated Corn Silk: Proximate Profile

**FIGURE 13:**
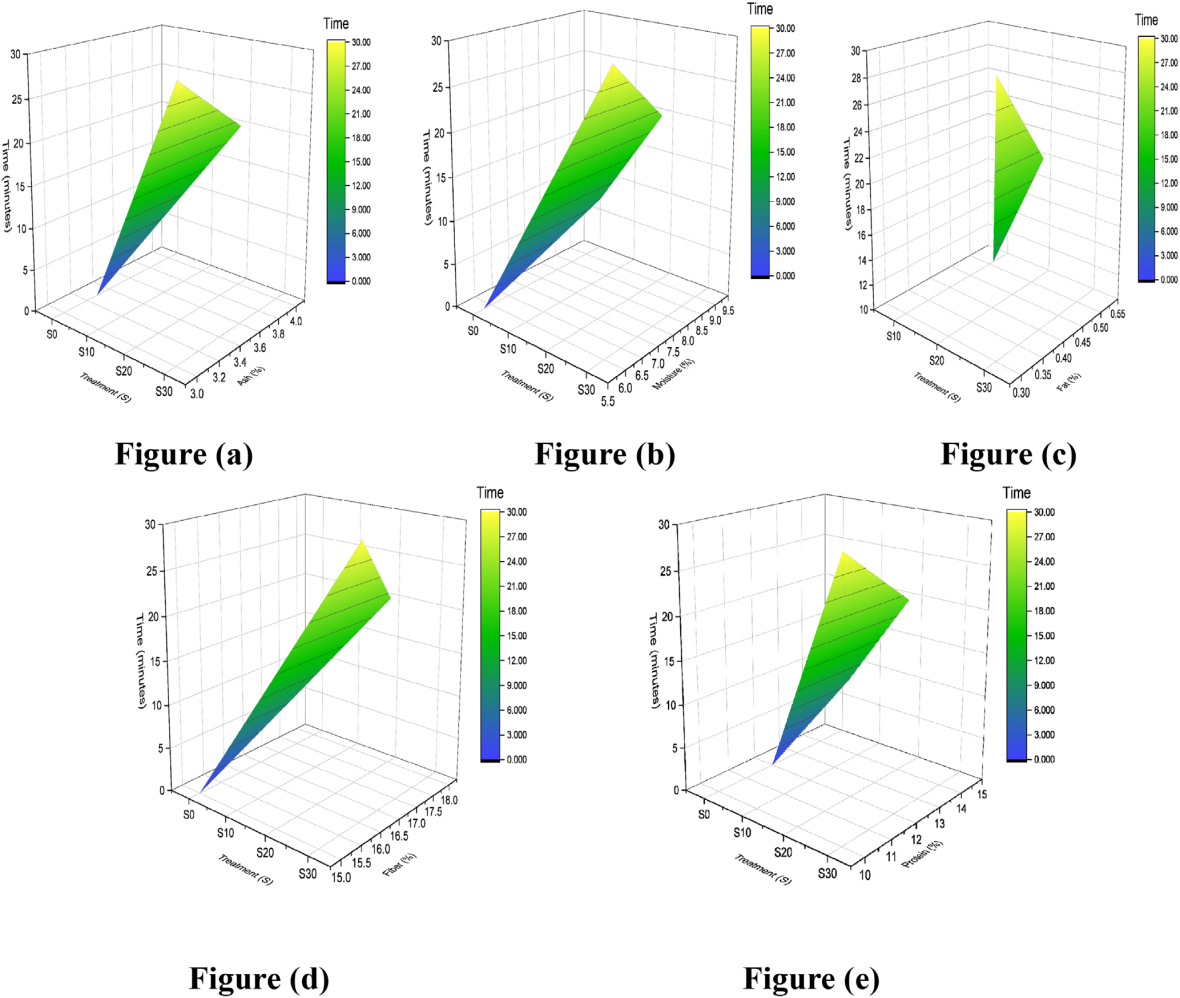
3D surface plot of silk: (a) ash, (b) moisture, (c) fat, (d) fiber, and (e) protein.

In the proximate profile of the corn silk control sample significant difference is observed as compared to S20 at a temperature of 40°C, as illustrated in Table 12. However, levels of ash, moisture, crude fat, crude fiber, and protein contents alter their concentration of 4.00% ± 0.08%, 9.10% ± 0.08%, 0.53% ± 0.01%, 18.00% ± 0.4%, and 14.40% ± 0.33%, respectively, elaborated by surface plots in Figure 13. The yield increased as the extraction time extended from 10 to 20 min. This trend can be attributed to the fact that ultrasound treatment promotes the disruption of corn silk cell walls, thereby enhancing both solubility and diffusion (Maran et al. 2013; Yadav et al. 2021). Considering these results, sonication demonstrates a distinct advantage as a non‐destructive processing technique, as it preserves essential fatty acids from degradation. It turns out that the average pore size, pore size with maximum ratio, surface porosity, and surface fractal dimension all rise nonlinearly as power and duration increase (Li et al. 2021). Many studies reported the same results; for instance, the ideal temperature for corn silk powder extraction was noted in the study to be 40°C, and the extraction process took 60 min (Praseptiangga et al. 2019). However, a study on barley grass observed by researchers reduced drying time by 14% and energy consumption by 19% when subjected to ultrasonic treatments (UT) for 10 min, and bathing materials were subjected to UT at varying power levels (10, 30, 45, and 60 W/L) (Cao et al. 2018). Some nutrients rose, and some declined when our results were compared to the literature. Therefore, the use of sonication can be considered an effective approach.

**TABLE 12:** Chemical composition of sonicated corn silk.

Parameter (%)	Control	S10	S20	S30
Ash	3.29 ± 0.1^b^	3.50 ± 0.08^b^	4.00 ± 0.08^a^	3.00 ± 0.01^c^
Moisture	5.80 ± 0.3^c^	8.30 ± 0.2^b^	9.10 ± 0.08^a^	6.20 ± 0.1^c^
Fat	0.45 ± 0.05^b^	0.51 ± 0.01^a^	0.53 ± 0.01^a^	0.30 ± 0.01^c^
Fiber	15.0 ± 0.7^d^	16.4 ± 0.3^b^	18.0 ± 0.4^a^	16.0 ± 0.6^bc^
Protein	12.0 ± 0.9^b^	13.6 ± 0.1^b^	14.4 ± 0.33^a^	10.0 ± 1.6^c^

*Note:* Values presented as means ± standard error, *n* = 3. Values within each column followed by different letters are significantly different at *p* ≤ 0.05. S = Corn silk; S0 = 0 min; S10 = 10 min; S20 = 20 min; S30 = 30 min.

### 3.10 Corn Silk Phytochemical Profile Under Sonication

**FIGURE 14:**
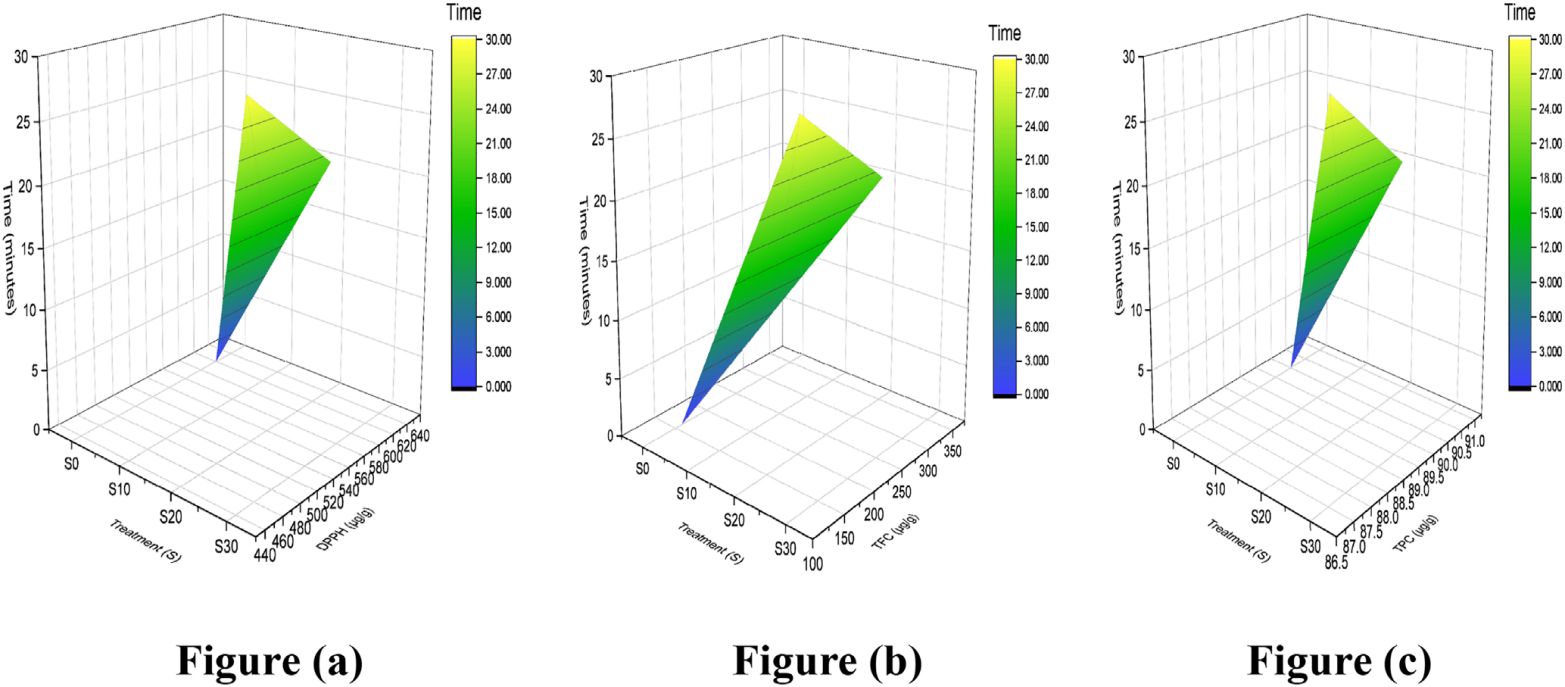
3D surface plot of the silk antioxidant profile: (a) DPPH, (b) TFC, and (c) TPC.

Depending on the time duration of sonication, Table 13 presents the statistically significant differences with *p*‐values below 0.05. Among the S0, S10, S20, and S30, all parameters were evaluated. The overall variation in concentration was observed within the 20%–30% range for DPPH, TFC, and TPC in S20, as further illustrated by the surface plots in Figure 14. It is justified by a study that reported the extraction of TFC from corn silk optimized under ultrasonic power of 500 W, with an extraction time of 20 min. Under these conditions, the extraction yield of TFC reached 1.13%. The high 467.59 μmol/L FRAP value suggests that the flavonoid extract from corn silk possesses significant antioxidant capacity at 20 min (Zheng et al. 2016). In another research, scientists studied the optimized process conditions utilizing the response surface method and a central composite design. The impacts of extraction factors on the TPC, TFC, and TAA of spinach roots were studied during the optimization process. The optimal extraction conditions of time (3 min), temperature (10°C), amplitude (55%), and ethanol concentration (20%) resulted in the highest levels of phenolic, flavonoid, and antioxidant activity: 5579.51 mg GAE kg^−1^ LW, 1824.34 mg CE kg^−1^ LW, and 20.79 mmol TE kg^−1^ LW (Ozcan and Damar 2023). This reinforces that ultrasonic time may vary on the basis of the structural and biological characteristics of the plant material.

**TABLE 13:** Antioxidant profile of sonicated corn silk samples.

Parameter (μg/g)	Control	S10	S20	S30
DPPH	600 ± 0.5^bc^	610 ± 0.1^ab^	620 ± 0.1^a^	450 ± 4.08^c^
TFC	160 ± 0.49^c^	240 ± 0.49^b^	350 ± 0.49^a^	120.0 ± 0.49^d^
TPC	90.0 ± 0.13^c^	90.3 ± 0.05^b^	90.8 ± 0.16^a^	87.0 ± 0.05^d^

*Note:* Values presented as means ± standard error, *n* = 3. Values within each column followed by different letters are significantly different at *p* ≤ 0.05. S = Corn silk; S0 = 0 min; S10 = 10 min; S20 = 20 min; S30 = 30 min.

### 3.11 Corn Silk Minerals Profile Altered by Sonication

**FIGURE 15:**
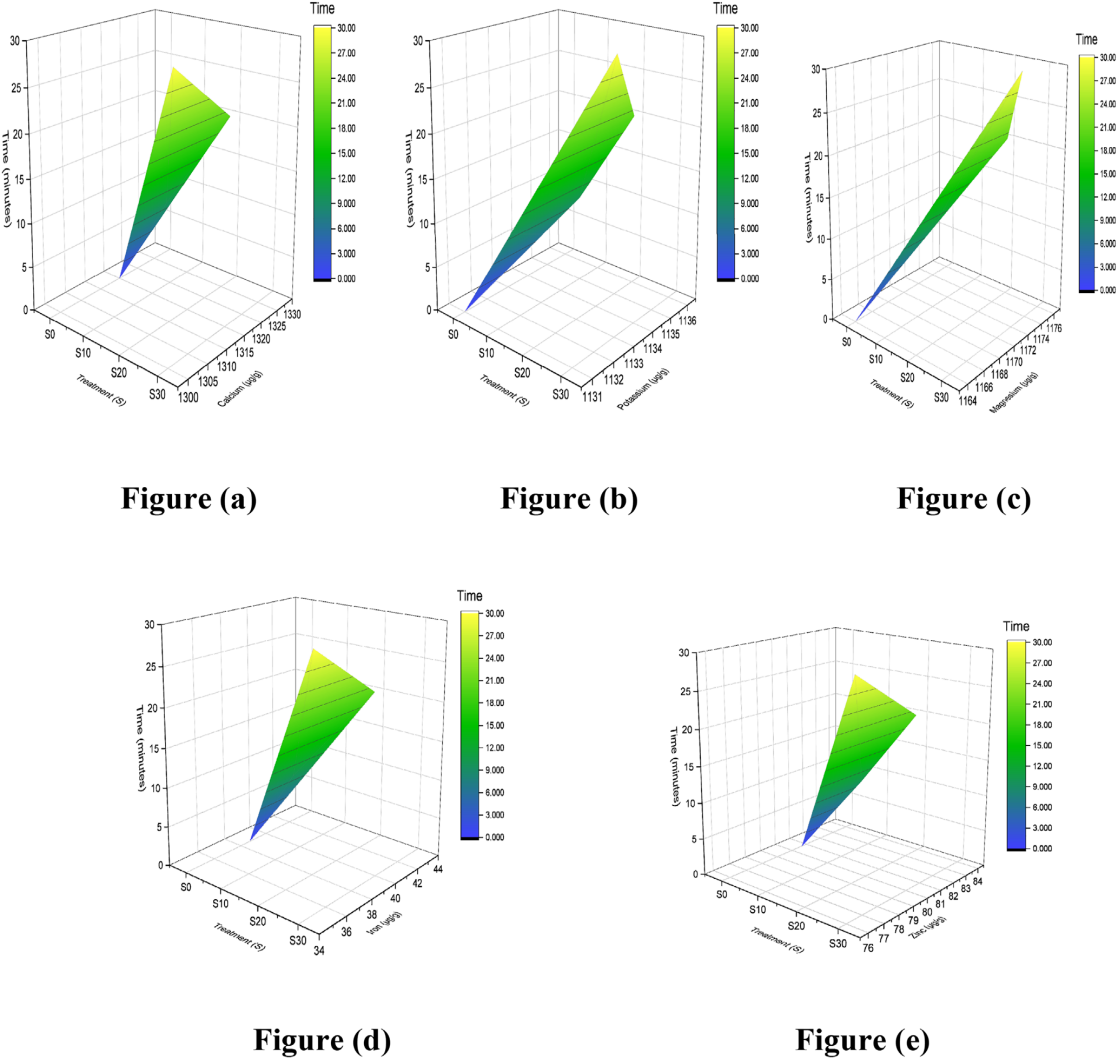
3D surface plot of minerals in silk: (a) calcium, (b) potassium, (c) magnesium, (d) iron, and (e) zinc.

The comprehensive analysis of corn silk revealed concentrations of Ca, Fe, Mg, K, and Zn as 1327.73 ± 0.09, 43.17 ± 0.47, 1176.47 ± 0.05, 1136.07 ± 0.09, and 83.63 ± 0.38 μg/g, respectively, along with their associated *p*‐values mentioned in Table 14. The temporal variation in mineral content is depicted in Figure 15. These findings are further supported by the characterization of corn silk extracts for antioxidant and physicochemical properties using different extraction techniques, including aqueous extraction, sonication‐assisted aqueous extraction, and ethanol extraction. Among these, sonication‐assisted aqueous extraction demonstrated enhanced mineral recovery, with Ca (mg/100 g) increasing from 245.67 ± 8.92 to 298.45 ± 10.23, Fe from 12.45 ± 0.45 to 15.78 ± 0.56, Mg from 156.78 ± 5.23 to 189.23 ± 6.45, K from 1245.67 ± 34.56 to 1456.78 ± 38.92, and Zn from 8.92 ± 0.34 to 10.67 ± 0.38 (Patel et al. 2025).

**TABLE 14:** Mineral composition of sonicated corn silk.

Parameters (μg/g)	Control	S10	S20	S30
Calcium	1315 ± 9.96^b^	1319 ± 0.47^b^	1327 ± 0.09^a^	1301 ± 0.05^c^
Iron	38.7 ± 1.96^b^	39.4 ± 2.45^b^	43.1 ± 0.47^a^	35.0 ± 0.05^c^
Magnesium	1164 ± 9.72^a^	1170 ± 0.05^a^	1176 ± 0.05^a^	1173 ± 0.47^a^
Potassium	1131 ± 4.93^a^	1135 ± 0.21^a^	1136 ± 0.09^a^	1133 ± 0.47^a^
Zinc	80.7 ± 1.31^b^	82.4 ± 0.08^a^	83.6 ± 0.38^a^	76.4 ± 0.05^c^

*Note:* Values presented as means ± standard error, *n* = 3. Values within each column followed by different letters are significantly different at *p* ≤ 0.05. S = Corn silk; S0 = 0 min; S10 = 10 min; S20 = 20 min; S30 = 30 min.

### 3.12 Corn Silk Vitamin Effect by Sonication

**FIGURE 16:**
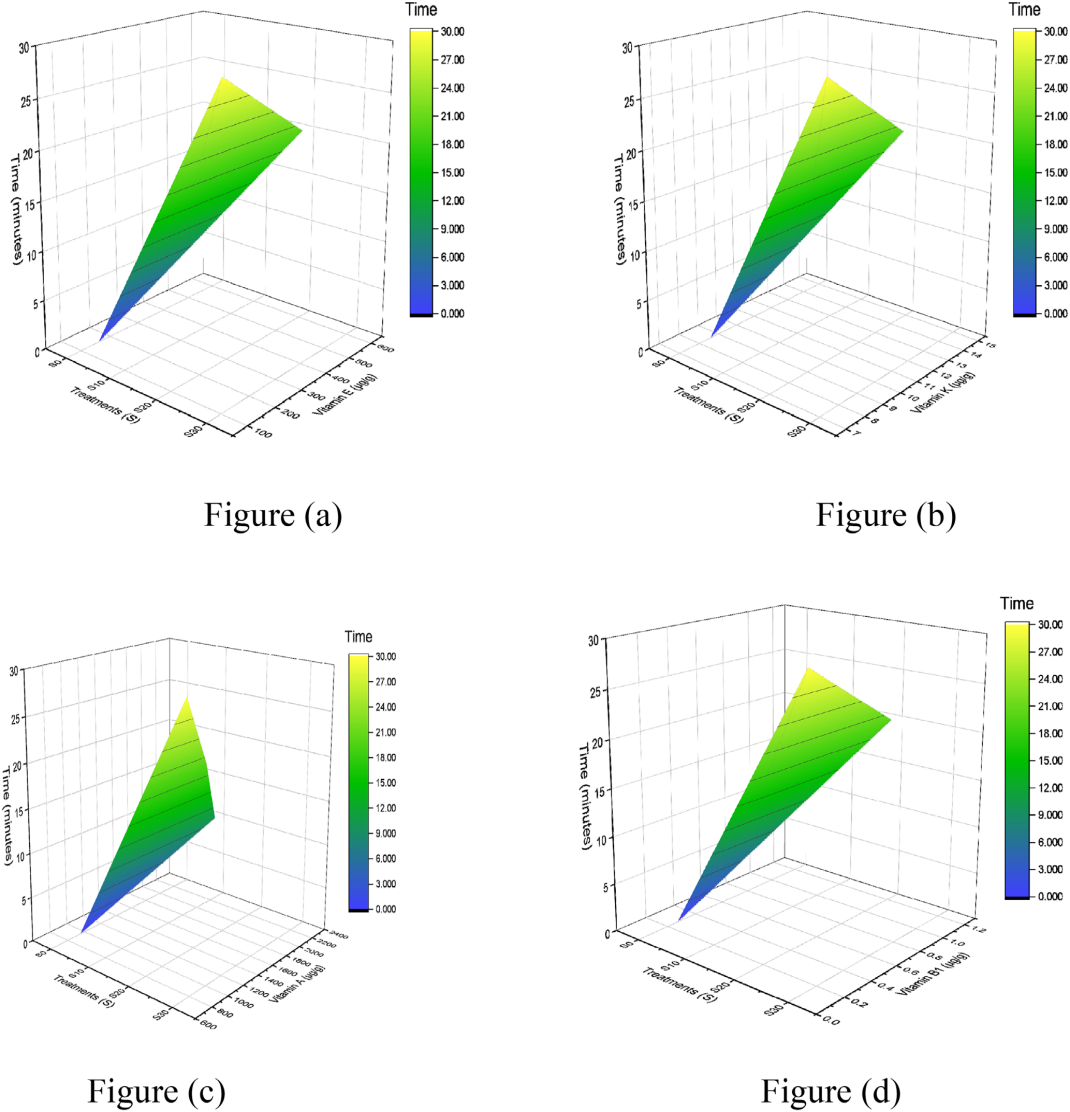
3D surface plot of silk vitamins: (a) vitamin E, (b) vitamin K, (c) vitamin A, and (d) vitamin B1.

The variations in the vitamin concentration in corn silk recorded with time are elaborated in Figure 16. Comparison of the corn silk control sample and other treatments with significant value at a constant temperature of 35°C–40°C, as shown in Table 15. The levels of vitamin E, A, K, and B1 in μg/g, 600 ± 0.09, 14.6 ± 0.4, 1560 ± 0.9, and 1.2 ± 0.09 were elaborated. A study reported a peak shift from 202 to 206 nm in red bean protein and lutein, leading to structural modifications in the complexes. Lutein (LU) is a naturally occurring carotenoid known for its hydrophobic nature and the presence of several unsaturated double bonds in its structure. Ultrasonic treatment at 20 min on 240 W works on the principle of emulsifying, which modifies the antioxidant properties of the complexes. It also modified their digestive behavior, increasing lutein release in the intestinal phase and enhancing its overall bio accessibility (Zhao et al. 2022). Our results also uniformly aligned with a study that reported the effect of ultrasound‐assisted solvent extraction (UASE) conditions on vitamin K1 extraction efficiency from iceberg lettuce, along with other plant materials like spinach, sprouts, in which sonication of lettuce for 20 min gave an increase in vitamin K yield from 8 to 15 (μg/g) (Bryshten et al. 2024). Vitamin E is a fat‐soluble vitamin composed of a terpenoid (prenyl or phytyl) tail and a chromanol head. Its terpenoid tail is a key structural feature that allows it to integrate into cell membranes and function as a fat‐soluble antioxidant. So ultrasonication (UAE) of celery and its enhanced yield of terpenoid justified the results because in that study, the ideal UAE parameters for terpenoids were 0.018 g/mL of sample‐to‐solvent, 10.60% water content, 37.28°C, 18.71 min extraction time, and 416.22 W of ultrasonic power under which the total terpenoid content was 76.22 ± 0.712 mg UE/g (Vo et al. 2025). Vitamin B1 content proven from another study which assessed the combined effects of ultrasonication parameters (sonication temperature [STemp]: 40°C, 50°C, and 60°C) and heating time (STime: 60 and 120 min) and drying techniques (air drying [AD], hot AD [HAD], microwave drying [MD], and freeze‐drying [FD]) on the extraction of phytochemicals from *Amaranthus hybridus* stem using natural deep eutectic solvent (NADES). The results showed that the TFC was lowest for AD combined with 60°C and highest for AD with 50°C ST at 60 min of sonication. In contrast, for 120 min of sonication, MD and AD at 50°C had the highest TFC. Although MD maintained vitamin C, and FD retained more vitamins, including thiamine, riboflavin, and niacin (Okonkwo et al. 2024).

**TABLE 15:** Vitamin profile of the sonicated corn silk sample.

Vitamins (μg/g)	Control	S10	S20	S30
Vitamin E	160 ± 0.04^a^	300 ± 0.09^b^	600 ± 0.09^a^	100 ± 1.3^d^
Vitamin K	8.6 ± 0.4^a^	11.6 ± 0.4^c^	14.6 ± 0.4^a^	7.3 ± 0.4^c^
Vitamin A	950 ± 0.4^c^	1160 ± 0.4^b^	1560 ± 0.9^a^	760 ± 0.4^c^
Vitamin B1	0.27 ± 0.04^c^	0.43 ± 0.04^c^	1.2 ± 0.09^a^	0.13 ± 0.04^c^

*Note:* Values presented as means ± standard error, *n* = 3. Values within each column followed by different letters are significantly different at *p* ≤ 0.05. S = Corn silk; S0 = 0 min; S10 = 10 min; S20 = 20 min; S30 = 30 min.

## 4 Principal Component Analysis

**FIGURE 17:**
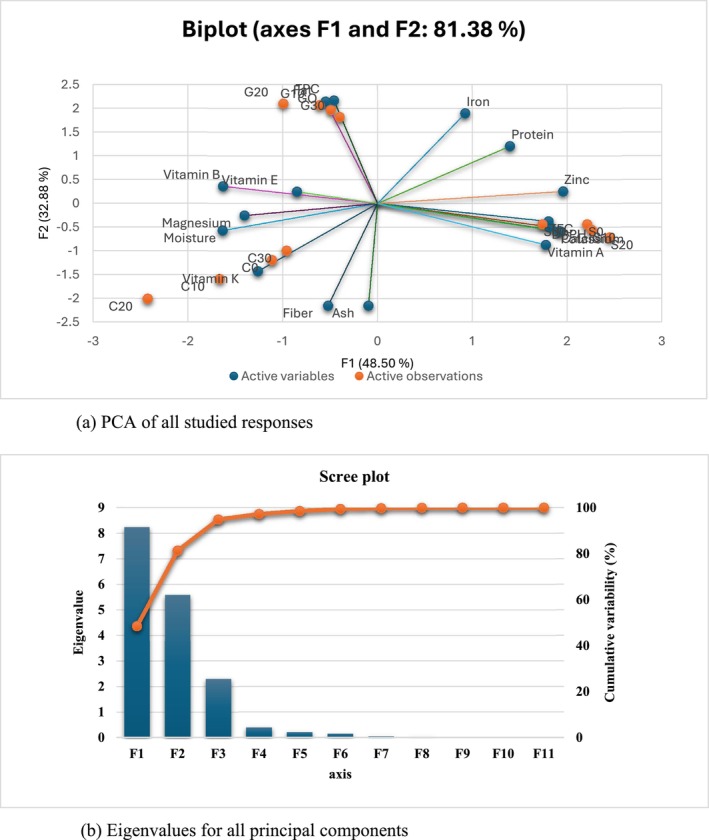
(a) PCA of all studied responses. (b) Eigenvalues for all principal components.

The provided component plot in rotated space illustrates the relationships between various nutritional and compositional variables (e.g., moisture, fiber, iron, ash, protein, zinc, TFC, DPPH, potassium‐calcium) across two principal components (Component 1 and Component 2). Component 1 accounts for 53.453% of the total variance, whereas Component 2 explains 38.550% along with maximum eigenvalues of 6.949 and 5.011, respectively, shown in Figure 17b. It cumulatively covers 92.003% of the dataset's variability. The remaining components contribute minimally (cumulative variance < 8%), suggesting that the first two components sufficiently capture the dominant patterns in the data. Within PC1 and PC 2 variables, ash, calcium, DPPH, fat, iron, magnesium, protein, potassium, TPC, TFC, zinc, and vitamins K and A show positive correlation, whereas B1 relates moderately. Moreover, fiber and moisture fall in PC2; Figure 17a illustrates a negative correlation. The PCA results demonstrate that sonication treatment significantly influenced the physicochemical properties of grains, cob, and silk, enhancing key nutritional and bioactive components. A strong positive correlation was observed with ash, fat, protein, essential minerals (calcium, magnesium, iron, potassium, zinc), antioxidant activity (DPPH, TPC, and TFC), and vitamins. However, ultrasound processing had a reducing effect on fiber and moisture content. These findings align with the earlier quantitative analysis and phytochemical characterization, confirming that sonication effectively modifies the compositional profile while maintaining consistency with prior observations.

## 5 Storage Analysis of Products

### 5.1 Total Moisture Counts

**FIGURE 18:**
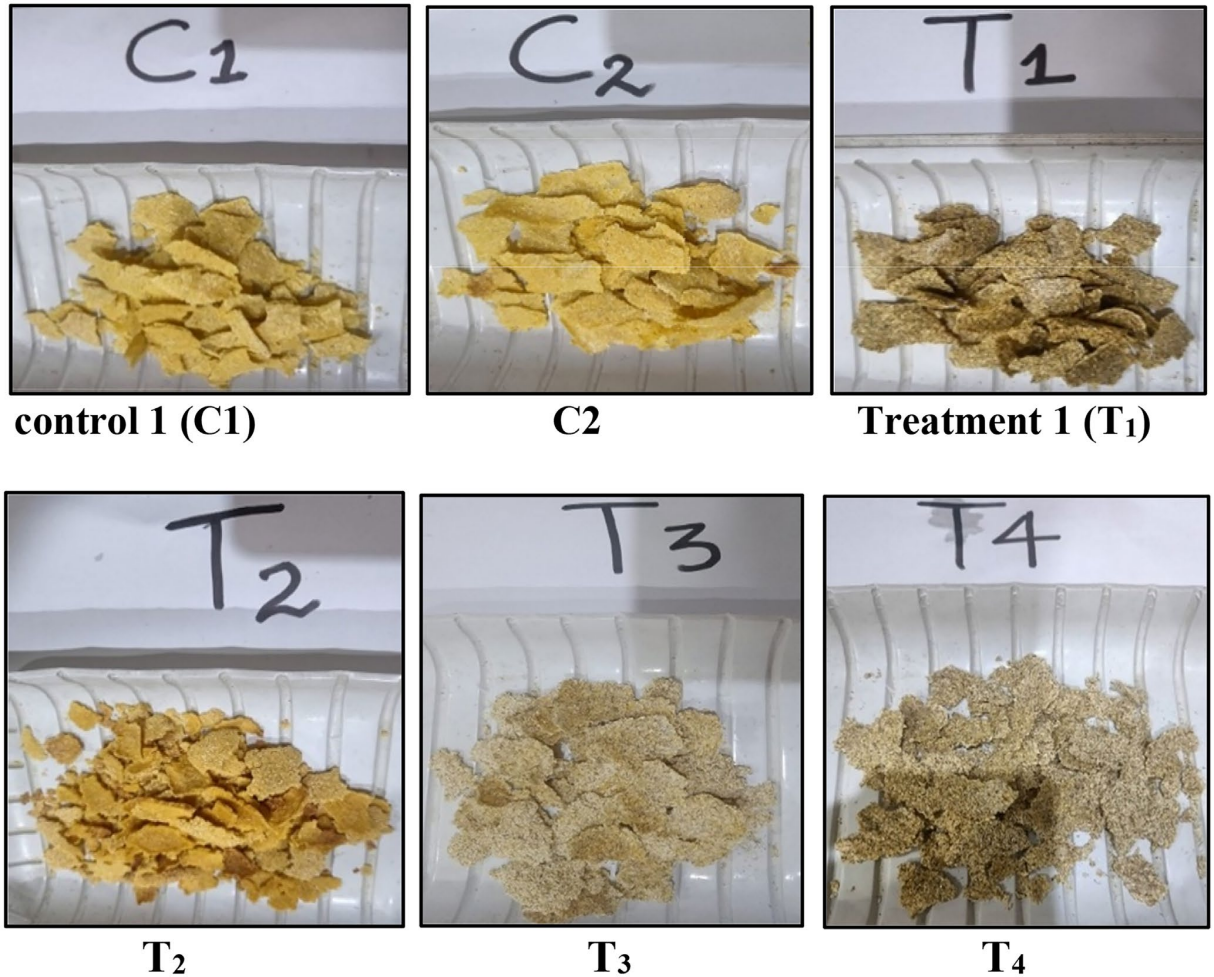
Flakes developed in different concentrations. *C*_1_ = 100% CGF; *C*_2_ = 100% SCGF; *T*_1_ = (70% SCGF + 10% SCF + 10% SSP + 10% QF); *T*_2_ = (50% SCGF + 30% SCF + 10% SSP + 10% QF); *T*_3_ = (30% SCGF + 50% SCF + 10% SSP + 10% QF); *T*_4_ = (10% SCGF + 70% SCF + 10% SSP + 10% QF).

**FIGURE 19:**
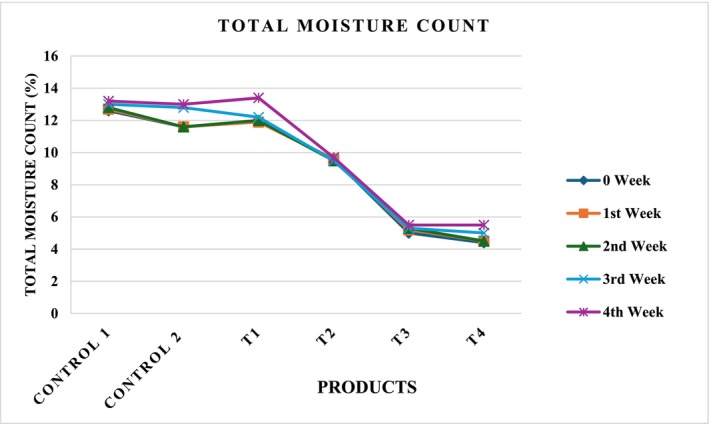
Four weeks count of total moisture in flakes.

Flakes developed with different concentrations, as shown in Figure 18, labeled as *C*_1_, *C*_2_, *T*_1_, *T*_2_, *T*_3_, and *T*_4_ were analyzed for the storage study. The effect of storage duration on the total moisture content of sonication‐pretreated corn flakes and their composite formulations is presented in Table 16. A progressive increase in moisture content was observed across all treatments during the four‐week storage period, although the magnitude of change varied with the formulation. For Control 1 (*C*_1_), the moisture content increased steadily from 12.6% at week 0 to 13.2% at week 4. This gradual rise was attributed to the hygroscopic nature of corn‐based products, which tend to absorb ambient moisture during storage. A similar trend was observed in Control 2 (*C*_2_), where moisture remained constant (11.6%) up to the second week but then increased sharply to 13.0% by week 4. The delayed moisture uptake in *C*_2_ suggests that treatment may have initially enhanced the resistance of the flakes to environmental humidity, although this effect diminished with extended storage. Among the blends, *T*_1_ exhibited a moderate but consistent increase in moisture content, rising from 11.9% to 13.4% over the storage period. The higher final value compared to both controls indicates that the composite formulation with moderate substitution allowed greater water‐binding capacity, possibly because of structural changes induced by sonication that enhanced porosity and moisture retention. In contrast, *T*_2_ displayed remarkable stability, with a narrow moisture content range of 9.5%–9.7% throughout the 4 weeks, as shown in Figure 19. More distinct patterns were observed in *T*_3_ and *T*_4_, which showed the lowest initial moisture contents (5.0% and 4.4%, respectively). Both treatments recorded only slight increments by week 4 (5.5%), indicating strong resistance to moisture absorption during storage. The lower values may be attributed to the reduced proportion of corn and increased incorporation of other components that possibly limited hygroscopicity. Overall, the results demonstrate that moisture uptake in sonicated flakes is influenced by both storage time and formulation ratio. Although controls and *T*_1_ showed significant increases in moisture, indicating higher susceptibility to ambient humidity, formulations with higher substitution levels (*T*_2_–*T*_4_) maintained relatively stable and lower moisture contents. These findings suggest that partial replacement of corn with alternative ingredients, especially at higher proportions, enhances the storage stability of flakes by minimizing moisture absorption. In a previous study, bananas treated with a combination of hot water (HW) and ultrasound (US) showed noticeably lower rates of decay incidence (65.63% ± 3.9%) and weight loss (18.62% ± 1.14%) in contrast to untreated (Alam et al. 2025).

**TABLE 16:** Total moisture counts in flakes.

Treatments (SCGF + SCF + SSP+ QF) %	0 Week	1st Week	2nd Week	3rd Week	4th Week
Control 1 (Untreated 100% corn)	12.6 ± 0.02^a^	12.7 ± 0.23^b^	12.8 ± 0.04^c^	13 ± 0.06^c d^	13.2 ± 0.01^d^
Control 2 (Treated 100% corn)	11.6 ± 0.7^a^	11.6 ± 0.9^a^	11.6 ± 0.1^a^	12.8 ± 0.02^c^	13 ± 0.01^d^
*T*_1_ (70 + 10 + 10 + 10)	11.9 ± 0.1^a^	11.9 ± 0.4^a^	12 ± 0.3^b^	12.2 ± 0.06^d^	13.4 ± 0.05^d^
*T*_2_ (50 + 30 + 10 + 10)	9.6 ± 0.03^a^	9.6 ± 0.02^a^	9.5 ± 0.05^a^	9.5 ± 0.04^b^	9.7 ± 0.06^d^
*T*_3_ (30 + 50 + 10 + 10)	5 ± 0.01^c^	5.2 ± 0.01^d^	5.3 ± 0.03^c^	5.3 ± 0.03^c^	5.5 ± 0.04^d^
*T*_4_ (10 + 70 + 10 + 10)	4.4 ± 0.09^a^	4.5 ± 0.02^a c^	4.5 ± 0.06^a c^	5 ± 0.07^d^	5.5 ± 0.09^d^

*Note:* Values within each column followed by different letters are significantly different at *p* ≤ 0.05. Values presented as means ± standard error, *n* = 3.

### 5.2 Total Bacterial Counts

**FIGURE 20:**
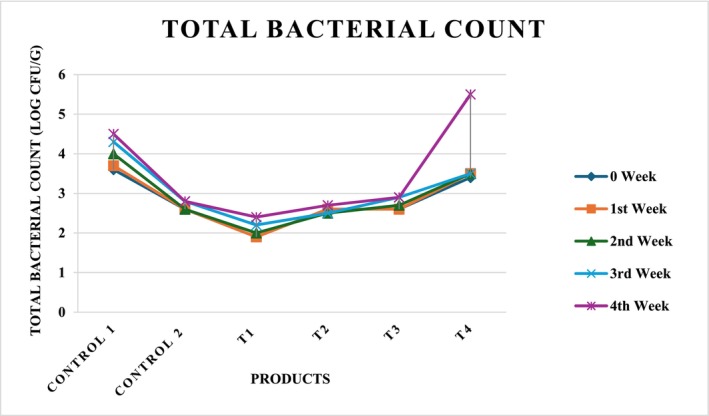
Four weeks total bacterial count in the flakes sample.

The microbial quality of cereal‐based flakes is a critical determinant of their shelf life and consumer safety. The results presented in Table 17 highlight the influence of sonicated corn, cob, and silk incorporation in flakes on the total bacterial counts over a 4‐week storage period. *C*_1_ exhibited the highest bacterial proliferation throughout the storage duration, with counts increasing steadily from 3.6 ± 0.2 log CFU/g at week 0 to 4.5 ± 0.1 log CFU/g at week 4. This continuous rise reflects the absence of antimicrobial intervention and confirms the susceptibility of untreated flakes to microbial growth during storage. By contrast, *C*_2_ demonstrated relatively stable bacterial counts (2.6–2.8 log CFU/g), indicating that processing treatment significantly suppressed microbial growth compared to the untreated control. *T*_1_ showed the lowest bacterial load across all storage intervals. Initial counts were 1.9 ± 0.1 log CFU/g at week 0 and increased moderately to 2.4 ± 0.02 log CFU/g by week 4. This minimal growth suggests that sonication treatment coupled with the compositional blend effectively inhibited microbial proliferation, likely because of structural modifications in the matrix and reduced water activity that limited microbial survival. Similarly, *T*_2_ and *T*_3_ exhibited controlled bacterial growth, with counts remaining below 3 log CFU/g throughout storage, as elaborated in Figure 20, thereby confirming the preservative effect of sonication and formulation adjustments. Conversely, *T*_4_ demonstrated a distinct microbial pattern. Although initial counts were comparable to *C*_1_, a sharp increase was observed after the third week, reaching 5.5 ± 0.01 log CFU/g by week 4, surpassing all other treatments. This higher susceptibility may be attributed to the higher quinoa concentration, which could provide a more favorable substrate for microbial growth because of its nutrient composition and higher residual moisture retention compared to corn‐dominant formulations. Overall, the findings indicate that sonication‐assisted processing markedly improved microbial stability compared to untreated controls. *T*_1_ emerged as the most effective treatment, maintaining bacterial counts at the lowest levels throughout storage, suggesting enhanced safety and prolonged shelf life. Food preservation can be achieved by using ultrasound as a processing aid to inactivate bacteria. However, protein and other chemicals in food matrices may be altered because of ultrasonication. These changes may be advantageous (lowering an unwanted flavor) or unfavorable (BA production). Because of this, it is crucial to assess the development of BA following the ultrasonic treatment of proteinaceous bulk. A crucial foodborne pathogen, *Listeria monocytogenes* (LM), is difficult to effectively manage during food preparation and storage because of its remarkable environmental adaptability and biofilm‐forming ability. By demonstrating that ultrasound in conjunction with capsaicin treatment significantly raises bacterial oxidative stress levels, increases cell membrane permeability, disrupts cellular architecture, and causes damage to bacterial DNA and proteins, ultimately resulting in bacterial death, the study clarifies the antibacterial mechanism of capsaicin as a sonosensitizer against LM (Yang et al. 2025). The significance of these results lies in their strong potential for practical application.

**TABLE 17:** Total bacterial counts in sonicated‐assisted flakes.

Treatments (SCGF + SCF + SSP + QF) %	0 Week	1st Week	2nd Week	3rd Week	4th Week
Control 1 (Untreated 100% corn)	3.6 ± 0.2^a^	3.7 ± 0.02^b^	4 ± 0.4^c^	4.3 ± 0.6^c d^	4.5 ± 0.1^d^
Control 2 (Treated 100% corn)	2.6 ± 0.07^a^	2.6 ± 0.08^a^	2.6 ± 0.01^a^	2.8 ± 0.02^c^	2.8 ± 0.01^d^
*T*_1_ (70 + 10 + 10 + 10)	1.9 ± 0.1^a^	1.9 ± 0.4^a^	2 ± 0.3^b^	2.2 ± 0.06^d^	2.4 ± 0.02^d^
*T*_2_ (50 + 30 + 10 + 10)	2.6 ± 0.03^c^	2.6 ± 0.02^c^	2.5 ± 0.05^a^	2.5 ± 0.04^a^	2.7 ± 0.05^d^
*T*_3_ (30 + 50 + 10 + 10)	2.6 ± 0.02^a^	2.6 ± 0.02^a^	2.7 ± 0.02^c^	2.9 ± 0.02^d^	2.9 ± 0.02^c^
*T*_4_ (10 + 70 + 10 + 10)	3.4 ± 0.04^b^	3.5 ± 0.03^c^	3.5 ± 0.05^a c^	3.5 ± 0.06^d^	5.5 ± 0.01^d^

*Note:* Values within each column followed by different letters are significantly different at *p* ≤ 0.05. Values presented as means ± standard error, *n* = 3.

### 5.3 Total Fungal Count in Flakes

**FIGURE 21:**
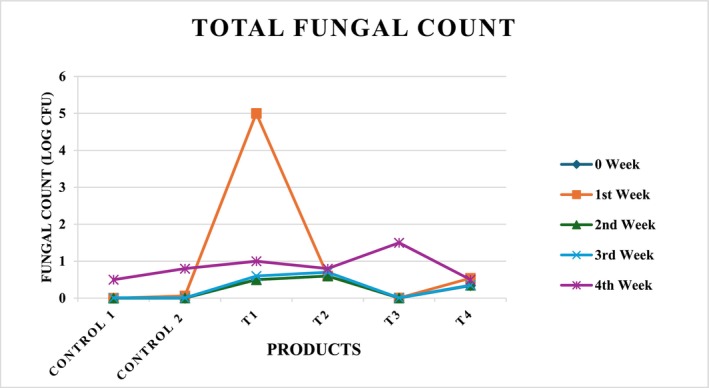
Four weeks total fungal count in flakes.

The results in Table 18 illustrate the effect of sonication‐assisted treatments on the fungal load of corn‐based flakes during 4 weeks of storage. Initially (0 week), all samples—including controls—showed negligible fungal counts, confirming good hygienic handling. Over time, fungal growth increased across treatments, with notable differences among formulations. The untreated control (*C*_1_) exhibited a gradual rise, reaching 0.5 log CFU by week 4, whereas the treated control (*C*_2_) increased to 0.8 log CFU, suggesting limited long‐term protection. Among sonicated samples, *T*_1_ and *T*_2_ remained stable for 2 weeks but rose to ~1.0 log CFU by week 4. *T*_3_ maintained low counts initially but reached 1.5 log CFU at the end of storage, indicating delayed but eventual fungal proliferation. In contrast, *T*_4_ showed minimal changes and the lowest final count (0.5 log CFU), demonstrating better inhibitory potential. Overall, sonication reduced initial fungal loads but did not fully prevent fungal growth during prolonged storage, as shown in Figure 21. The efficacy appeared formulation‐dependent, supporting the need for combined preservation strategies such as moisture optimization, natural antifungals, or modified atmosphere packaging to enhance shelf stability. The results align with previous reports where ultrasound‐assisted processing not only reduced moisture content, microbial contamination, and fungal content but also induced structural modifications that impaired bacterial survival during storage (Jasmi et al. 2019). Glutinous rice's soaking process promotes the development and spread of germs, which can quickly lead to issues with food safety. The bactericidal effect of ultrasound on glutinous rice during soaking and its influence on physicochemical properties of starch and quality characteristics of sweet dumpling were investigated. The overall bacterial count dropped by 2.04 log CFU/g after 30 min. Additionally, ultrasonic treatment breaks down the grain structure of glutinous rice starch, causing dents and cracks to form on the surface. This also increases the amount of amylose in the starch, improves its expansion, decreases its relative crystallinity and short‐range order, and changes its gelatinization properties (Wang, Wang, Liu, et al. 2024). These findings indicate that the incorporation of sonicated corn, cob, and silk into breakfast flakes significantly contributes to enhancing their storage stability.

**TABLE 18:** Total fungal count in flakes.

Treatments (SCGF + SCF + SSP + QF) %	0 Week	1st Week	2nd Week	3rd Week	4th Week
Control 1 (Untreated 100% corn)	0.001 ± 0.02^a^	0.001 ± 0.2^b^	0.001 ± 0.04^c^	0.01 ± 0.06^c d^	0.5 ± 0.01^d^
Control 2 (Treated 100% corn)	0.00 ± 0.7^a^	0.06 ± 0.8^a^	0.006 ± 0.01^a^	0.007 ± 0.02^c^	0.8 ± 0.01^d^
*T*_1_ (70 + 10 + 10 + 10)	0.5 ± 0.01^a^	05 ± 0.04^a^	0.5 ± 0.03^b^	0.6 ± 0.6^d^	1 ± 0.2^d^
*T*_2_ (50 + 30 + 10 + 10)	0.6 ± 0.3^c^	0.6 ± 0.2^c^	0.6 ± 0.5^a^	0.7 ± 0.4^a^	0.8 ± 0.5^d^
*T*_3_ (30 + 50 + 10 + 10)	0.006 ± 0.2^a^	0.006 ± 0.2^a^	0.006 ± 0.2^c^	0.009 ± 0.2^d^	1.5 ± 0.2^c^
*T*_4_ (10 + 70 + 10 + 10)	0.34 ± 0.04^b^	0.54 ± 0.3^c^	0.35 ± 0.5^a c^	0.35 ± 0.6^d^	0.5 ± 0.01^d^

*Note:* Values within each column followed by different letters are significantly different at *p* ≤ 0.05. Values presented as means ± standard error, *n* = 3.

## 6 Conclusion

The present study clearly established 20 min sonication at 40°C as the optimal processing condition, resulting in marked improvements in nutritional and functional attributes of corn grain, cob, and silk fractions. This treatment significantly enhanced proximate composition, particularly protein, fiber, and ash, alongside antioxidant capacity (DPPH) and key micronutrients, notably calcium and vitamin A. Importantly, incorporation of the optimized formulation (50% sonicated corn grain flour, 30% sonicated cob flour, 10% sonicated silk powder, and 10% quinoa flour) produced composite flakes with improved storage stability and microbial safety for up to 14 days, without compromising product quality. These results demonstrate the dual benefit of sonication in simultaneously improving nutritional value and shelf stability. Overall, the findings confirm that sonication is an effective, green, and non‐thermal technology for valorizing corn‐based agro‐industrial by‐products into functional foods. By enhancing nutrient density, antioxidant potential, and product stability, this approach supports sustainable food processing and circular economy principles. Future research should focus on process optimization, broader application to other cereal by‐products, and evaluation of industrial‐scale feasibility.

## Author Contributions

**Hina Niaz:** writing – original draft, formal analysis, and data curation. **Amna Javed:** visualization, data curation, conceptualization, and investigation. **Rida Niaz:** writing – review and editing.

## Funding

The authors have nothing to report.

## Ethics Statement

The authors have nothing to report.

## Conflicts of Interest

The authors declare no conflicts of interest.

## Data Availability

The data that support the findings of this study are available on request from the corresponding author. The data are not publicly available due to privacy or ethical restrictions.
